# LncRNA GAS5/miR-137 Is a Hypoxia-Responsive Axis Involved in Cardiac Arrest and Cardiopulmonary Cerebral Resuscitation

**DOI:** 10.3389/fimmu.2021.790750

**Published:** 2022-01-11

**Authors:** Wang Jing, Xie Tuxiu, Long Xiaobing, Jiang Guijun, Kang Lulu, Jiang Jie, Ye Lu, Zhan Liying, Xiong Xiaoxing, Lyu Jingjun

**Affiliations:** ^1^ Department of Emergency, Renmin Hospital of Wuhan University, Wuhan, China; ^2^ Department of General Practice, Renmin Hospital of Wuhan University, Wuhan, China; ^3^ Department of Critical Care Medicine, Renmin Hospital of Wuhan University, Wuhan, China; ^4^ Department of Neurosurgery, Renmin Hospital of Wuhan University, Wuhan, China

**Keywords:** neuroinflammation, exosome, microglia, astrocyte, crosstalk, cardaic arrest, cardiopulmonary cerebral resuscitation

## Abstract

**Background:**

Cardiac arrest/cardiopulmonary resuscitation (CA/CPR) represents one of the devastating medical emergencies and is associated with high mortality and neuro-disability. Post-cardiac arrest syndrome (PCAS) is mechanistically ascribed to acute systemic ischemia/reperfusion(I/R) injury. The lncRNA/microRNA/mRNA networks have been found to play crucial roles in the pathogenesis of the hypoxia-responsive diseases. Nonetheless, the precise molecular mechanisms by which lncRNA/miRNA/mRNA axes are involved in the astrocyte–microglia crosstalk in CA/CPR have not been fully elucidated.

**Methods:**

We collected and purified the exosomes from the blood of CA/CPR patients and supernatant of OGD/R-stimulated astrocytes. On the basis of microarray analysis, bioinformatic study, and luciferase activity determination, we speculated that lncRNA GAS5/miR-137 is implicated in the astrocyte–microglia crosstalk under the insult of systemic I/R injury. The regulation of lncRNA GAS5/miR-137 on INPP4B was examined by cellular transfection in OGD/R cell culture and by lateral ventricle injection with miR-137 agomir in CA/CPR mice model. Flow cytometry and immunofluorescence staining were performed to detect the microglial apoptosis, M1/M2 phenotype transformation, and neuroinflammation. Neurological scoring and behavior tests were conducted in CA/CPR group, with miR-137 agomir lateral-ventricle infusion and in their controls.

**Results:**

In all the micRNAs, miR-137 was among the top 10 micRNAs that experienced greatest changes, in both the blood of CA/CPR patients and supernatant of OGD/R-stimulated astrocytes. Bioinformatic analysis revealed that miR-137 was sponged by lncRNA GAS5, targeting INPP4B, and the result was confirmed by Luciferase activity assay. qRT-PCR and Western blotting showed that lncRNA GAS5 and INPP4B were over-expressed whereas miR-137 was downregulated in the blood of CA/CPR patients, OGD/R-stimulated astrocytes, and brain tissue of CA/CPR mice. Silencing lncRNA GAS5 suppressed INPP4B expression, but over-expression of miR-137 negatively modulated its expression. Western blotting exhibited that PI3K and Akt phosphorylation was increased when lncRNA GAS5 was silenced or miR-137 was over-expressed. However, PI3K and Akt phosphorylation was notably suppressed in the absence of miR-137, almost reversing their phosphorylation in the silencing lncRNA GAS5 group. Then we found that GAS5 siRNA or miR-137 mimic significantly increased cell viability and alleviated apoptosis after OGD/R injury. Furthermore, over-expression of miR-137 attenuated microglial apoptosis and neuroinflammation in CA/CPR mice model, exhibiting significantly better behavioral tests after CA/CPR.

**Conclusion:**

LncRNA GAS5/miR-137 may be involved in the astrocyte–microglia communication that inhibits PI3K/Akt signaling activation *via* regulation of INPP4B during CA/CPR.

## Introduction

Cardiac arrest/cardiopulmonary resuscitation (CA/CPR) is a devastating medical emergency with an extremely low short-term survival rate (1–3). In Europe, 300,000 cardiac arrests occur annually, and more than 85% of CA cases result in death (4). Cardiac arrest associated central nervous injury is the main cause of death in patients resuscitated from cardiac arrest, and the main cause of long-term disability in those who survive the acute phase of resuscitation (5, 6).

The cases of cardiac arrest due to stroke and cerebral trauma or tumor account for approximately 15–20% of all cardiac arrest patients (7). The most cardiac arrests are not of cerebral origin but are caused by extra-brain factors, such as arrhythmia, heart failure, respiratory failure, septic shock, severe hydro-electrolyte disequilibrium, among others (4). Therefore, Post Cardiac Arrest Syndrome (PCAS) is a typical condition in which extra-brain damage leads to extremely serious injury to the brain (8). Cardiac arrest persisting for 8 min results in irreversible damage to the central nervous system (CNS), which poses a seemingly insurmountable challenge for clinicians.

So far, mechanistically, PCAS is generally considered to be an acute systemic ischemia (no-flow)/reperfusion (low-flow) injury, which takes place sequentially during cardiac arrest, resuscitation, and the early phase of return of spontaneous circulation (ROSC) (8). Upon reperfusion of the cerebral vessels following systematic ischemia, the innate immune system triggers an inflammatory response characterized by activation of astrocytes under hypoxia/ischemia. Subsequently, the resident microglia/macrophages are activated and secrete pro-inflammatory cytokines, such as interleukin 6, interleukin 1-β, and chemokines, thereby aggravating damage to the nervous system (2).

Mounting evidence showed that exosomes, a type of extracellular vesicles released by astrocytes, act as a signaling transmitter among astrocytes, microglia, and neurocytes (9–11). By transporting protein or/and non-coding RNAs, exosomes play a pivotal role in triggering and regulating neuro-immune responses *via* adjacent and remote micro-communications. Current research has shown that the non-coding RNA contained in exosomes was mainly composed of long non-coding RNAs (lncRNAs), circular RNAs (circRNAs), and microRNAs (miRNAs) (11–14). Multiple studies have revealed that lncRNAs could exert negative regulatory effects on specific miRNAs, and miRNAs bound to the 3’-end non-coding region (3’-UTR) of mRNA through specific base complementary pairing to inhibit mRNAs transcription and translation, thereby negatively modulating the expression of a variety of mRNAs (15–18).

Regulatory networks among lncRNAs/miRNAs/mRNAs have been a subject of active investigations in many fields, such as immune regulation (19), neurodegeneration-related disorders (20, 21), and traumatic brain injury (9, 22), stroke (23), so on. These differential expressions of genes between injured tissues and their healthy counterparts can be used as diagnostic markers, research targets, or indicators of therapeutic interventions (24–26). However, studies focusing on exosomes in the astrocyte–microglia crosstalk under cardiac arrest are scanty. The exact roles of lncRNA/miRNA/mRNA axis in CA/CPR have not been fully elucidated.

In this study, an OGD/R cell model was constructed to investigate the microarray of astrocyte-derived exosomes, their effect on apoptosis and transformation of microglia phenotypes. Moreover, based on the miRNA-seq analysis, literature research (27), and bioinformatic study, a crucial LncRNA–microRNA–mRNA axis that regulates the course of acute systemic ischemia/reperfusion injury was identified. By gain-of-function and loss-of-function experiments, we specifically examined how the LncRNA GAS5/miR-137/INPP4B axis is involved in the OGD/R-stimulated astrocyte–microglia crosstalk. Of note, our miRNA-seq results with blood samples from CA/CPR patients were consistent with those of OGD/R cell experiments.

The expression of lncRNA growth arrest specific 5 (lncRNA GAS5) was upregulated and miR-137 was largely downregulated in response to hypoxia, not only in strocyte–microglia cell culture but also in blood samples from CA/CPR patients. Silencing the lncRNA GAS5 promoted the activation of the PI3K/AKT-mediated microglia apoptosis pathway, possibly by sponging miR-137 under OGD/R injury. Moreover, the function of the miR-137 was further investigated in a mouse CA/CPR model *in vivo* to explore a therapeutic target for CA/CPR-associated acute brain injury. In this study, we demonstrated that lncRNA GAS5/miR-137 was a hypoxia-responsive axis involved in the astrocyte–microglia crosstalk and could inhibit PI3K/Akt signaling pathway *via* regulating INPP4B in response to acute systematic ischemia/reperfusion associated neuro-injury.

## Materials and Methods

### Blood Sample Collection From Cardiac Arrest Patients and Ethics Considerations

The project was approved by the Research Ethics Board of Renmin Hospital of Wuhan University (Approval Number WDRY2020-KS032), Wuhan, China. We fully abide by all the protocols approved by the Board, and the all procedures complied with the principles of NMPA/GCP and the Declaration of Helsinki.

Since all patients in this study were in comatose state or/and dying, they were unable to respond to the questions or requests. Alternatively, their close relatives were informed about the trial and they signed the informed consent. If close relatives were not available, during the screening process of the study, the subject was included as an emergency procedure by the Emergency physician, which is in compliance with the applicable law of China. Informed consent exemption was approved by the same Board (Approval Number WDRY2020-KS032) as required.

The blood samples were taken from 12 h after ROSC from cardiac arrest patients who were hospitalized in the Emergency Department or/and Critical Care Medicine Department of Renmin Hospital of Wuhan University from 2020 to 2021. The data of CA patients are detailed in **Supplementary Table 1**.

### Murine Model of Cardiac Arrest/Cardiopulmonary Resuscitation (CA/CPR)

Animal experiments were approved by the Laboratory Animal Welfare & Ethics Committee of Renmin Hospital of Wuhan University (Approval Number WDRY IACUC 20210510), Wuhan, China. All animal experiments were performed in according to the procedures of Wuhan University and the National Institutes of Health Guidelines for the Care and Use of Laboratory Animals, Wuhan, China. Adult male C57BL/6 mice (aged 3–4 months, weighing 26–30 g) had *ad libitum* access to food and water in a room maintained on a 14:10 hour light/dark cycle.

All experimental animals were randomly divided into groups using Quickcalcs. CA/CPR operation was performed as previously described (28–31). In brief, after tracheal intubation, 1.5% isoflurane was given to maintain anesthesia before cardiac arrest. Electrocardiogram (ECG) and body core temperature were monitored during the entire procedure. After 30 μl of 0.5 mol/L potassium chloride (KCl) was infused to induce asystole, the mechanical ventilation was turned off.

Approximately 8.5 min after cardiac arrest, the brain heating system was removed, mechanical ventilator with pure oxygen was turned on, and 100 μl epinephrine (32 μg/ml) was administered and it was continuously infused at 25 μl/min. Manual chest compression was conducted until return of spontaneous circulation (ROSC). If ROSC could not be achieved within 3 min, cardiopulmonary resuscitation was terminated. In the sham group, anesthesia was induced with 5% isoflurane. After tracheal intubation, mice were given 1.5–1.7% isoflurane for about 20 min.

### Intracerebroventricular Infusion of miR-137-5p

miR-137-5p agomir and its negative control were dissolved and mixed according to the manufacturer’s instructions (RiboBio, China). After 15 min of reaction at room temperature, they were injected into lateral ventricle. A Hamilton syringe (Gaoge, China) was inserted at 0.5 mm posterior and 1.0 mm lateral to the bregma and 3.0 mm ventral to the skull under the guidance of a stereotaxic instrument (RWD Life Science). A single dose of miR-137-5p agomir (5 nM) was infused into the lateral ventricle 20 min before cardiac arrest.

### Neurologic Scoring and Behavioral Tests

Researchers who were blinded to experimental grouping did the neurologic scoring and behavioral tests.

*Neurologic scoring.* A 9-point scoring system, a well-established neurologic testing system designed to detect motor deficits in the rat, was modified and used to assess overall neurologic deficits in mice (32). The final score ranked from zero to nine, with normal mice being awarded 9 points and the severely injured ones 0 point.

*Open field test.* On day 3 after CA/CPR, mice were transferred to a quiet environment for an open field test. First, the mice were kept in their home cage for 1 h to familiarize with the environment before the test. Then, the mouse was placed in the center of the open field box (50 × 50 × 50 cm, CleverSys Inc, Reston, VA, USA), and allowed to explore for 10 min while being recorded by an overhead camera (28). Mouse behaviors were analyzed by the automated tracking system TopScan (CleverSys).

*Rotarod.* Rotarod was performed as previously described (29). Briefly, all animals were trained on daily basis for 3 days before CA/CPR surgery. Mice were gently placed on the rotarod, and the rotation speed was ramped up to 40 rpm within 5 min. The time from stay on the rotating disk to fall-down was recorded. The average of the three experimental results was taken as the final experimental data.

### Quantitative Reverse Transcription-Polymerase Chain Reaction (qRT-PCR)

In brief, the total RNA of the brain tissues or cultured cells was extracted using the TRIzol reagent (Invitrogen, Carlsbad, CA, USA), and was then used to generate cDNA samples with the PrimeScriptTM RT Reagent Kit (Thermo, USA) by following the manufacturer’s instructions.

The SYBR GREEN Mix (Invitrogen, Waltham, MA USA) reaction system was employed for qRT-PCR along with the forward primer, the reverse primer, and cDNA. U6 snRNA (MQP-0201, RiboBio, China) served as the internal control of miR-137-5p (MQP-0101, RiboBio, China), and glyceraldehyde 3-phosphate dehydrogenase (GAPDH) was used as the internal control of other mRNAs. RNA levels were assayed using the 2^−ΔΔCt^ method for relative expression. All primers used in this study are listed in **Supplemental Table 2**.

### Western Blotting

The standard Western blot method was based on the previous literature (33). Hippocampus tissues or cultured cells were quickly dissected out on ice, and lysed in radioimmuno-precipitation assay (RIPA) buffer. BCA kit was used to determine protein concentration. The proteins were then separated by sodium dodecyl sulfate-polyacrylamide gel electrophoresis (SDS-PAGE) and transferred to a polyvinylidene fluoride membrane. Subsequently, the membrane was incubated with diluted primary antibodies overnight at 4°C. Primary antibodies used are listed in **Supplemental Table 3**. The signal intensity was quantitatively determined using ImageJ (NIH, Bethesda, MD, USA).

### Northern Blotting

miR-137-5p agomir and its negative control were infused into the lateral ventricle. Their mRNA expression was detected by Northern blot analysis. Briefly, RNA samples were electrophoresed on agarose gel, and transferred onto nitrocellulose filter membrane. Hybridization was done at 42°C in 7% SDS/0.2 mol/L Na_2_PO_4_ (pH 7.0) for 16 h. Membranes were washed at 42°C, twice with 2× standard saline phosphate [0.18 mol/L NaCl/10 mmol/L phosphate (pH 7.4)], 1 mmol/L EDTA (saline-sodium phosphate-EDTA, SSPE), and 0.1% SDS and twice with 0.2× SSPE/0.1% SDS. An oligonucleotide complementary to the U6 RNA was used to normalize expression levels. Blots were stripped in boiling 0.1% SDS for 10 min before rehybridization.

### Immunofluorescence Staining

The cells seeded on the slide were plated onto slides. After the cells were fixed and washed with PBS, and slides were incubated with GFAP, Iba1, CD9, CD63, Arg1 or iNOS antibodies overnight at 4°C. After repeated PBS washing, the slides were incubated with corresponding secondary antibodies for 1 h at room temperature in the dark. After several washes with PBS, the slides were incubated with DAPI for 3 min and then mounted with anti-fluorescence quenching sealing agent. After mounting, immunofluorescence images were taken under an Olympus inverted fluorescence microscope (Olympus, Tokyo, Japan), and the percentages of positive cells were recorded by two researchers who were blinded to study design by using Image J. In **Supplemental Table 3**, primary antibodies are shown.

### Flow Cytometry

Briefly, mice were deeply anesthetized with isoflurane, and transcardially perfused with ice-cold PBS. After dissection of olfactory bulb and epencephalon tissues, brain tissues were then cut into small pieces in ice-cold DMEM medium, and digested with Collagenase D (1 mg/ml) and DNAse I (0.1 mg/ml) (Sigma-Aldrich, St. Louis, MO, USA) for 45 min at 37°C. Cells were then filtered through a 70-μm cell strainer, and re-suspended in a 70%/37% Percoll gradient (GE Healthcare Life Sciences, Pittsburgh, PA, USA). After centrifugation at 500*g* without braking, the immune cells at interphase were harvested. After washing with 25 ml PBS, cell pellets were re-suspended in 200 μl PBS containing 1% fetal calf serum. Cells were counted on a hemocytometer after staining with trypan blue. Single-cell suspensions were then adjusted to approximately 1 × 10^6^ cells in 100 μl RPMI 1640 containing 2% fetal calf serum. After incubation with Fc receptor blocking solution for 10 min, cells were immunostained with different surface antibodies (listed in **Supplemental Table 3**). In brief, microglia/macrophages and neutrophils were stained with the following antibodies: CD45-FITC (103107), CD11b-PE/cy7 (101215), F4/80-APC (157305), Ly6G-APC/cy7 (127624). LIVE/DEAD fixable violet kit (L34963A; Invitrogen) was used to stain dead cells. Flow cytometry data were acquired on FACS Canto (BD Biosciences, San Jose, CA, USA) and analyzed using FlowJo software package.

### Culture of Primary Astrocytes

Primary astrocytes were obtained and purified on days 1 to 2 after the birth of C57/BL6 mice as previously described (9, 34). Briefly, the cerebral cortex of suckling mouse was taken out, minced into 1 mm^3^ pieces and placed in pre-cooled PBS. After digestion with 0.25% trypsin and DNase for 10 min, the cell suspensions were passed through a 70-μm cell strainer (BD Falcon), and the digestion was ended with Dulbecco’s modified Eagle’s medium (DMEM) containing 10% fetal bovine serum (FBS) and 1% penicillin/streptomycin. These cells were collected by centrifugation and inoculated into culture flasks. After culture for about 10–14 days, microglia in the astrocytes were removed by shaking at 220 rpm for 40 min, and oligodendroglia in the remaining mixed glial cells were removed by shaking at 220 rpm for 18 h. As a result, the remaining cells in the flask were astrocytes (purity >90%). Astrocytes were cultured in DMEM for subsequent experiments.

### Construction of Oxygen-Glucose Deprivation/Reperfusion (OGD/R) Cell Model

Before inducing OGD/R injury, cells were in the logarithmic growth phase. In brief, the cells were cultured in DMEM (without glucose) (Genom, Hang Zhou),and then placed into three gas incubators (Binder, CB160, Germany) with 1% O_2_, 5% CO_2_, and 94% N_2_ for 4 h at 37°C to imitate Oxygen-Glucose Deprivation. The cells in DMEM (with glucose) were placed in normoxic conditions (37°C, 5% CO_2_) for 8 h (Oxygen-Glucose Restoration). The control groups were incubated in DMEM with normal glucose for 12 h in an atmosphere of 95% air and 5% CO_2_.

### Transfection of the miR-137-5p Mimic and miR-137-5p Inhibitor Into Cells

The miR-137-5p mimic, miR-137-5p inhibitor and their corresponding negative control (mimic NC and inhibitor NC) were purchased from RiboBio (China). They were dissolved and mixed according to the manufacturer’s protocol. The BV2 microglial cell were transfected with 100 nM aliquots of either the miR-137-5p mimic or miR-137-5p inhibitor using RiboFECT™ CP (RiboBio, China) by following the manufacturer’s instructions.

### Apoptosis Assay

Cells in above-mentioned groups were harvested for apoptosis assay. The samples were immediately re-suspended in the binding buffer and subsequently stained with 5 ml of APC Annexin V and 5 ml of 7-AAD according to the kit instructions (BD Biosciences, USA). The mixture was incubated at room temperature for 15 min, and was immediately subjected to the apoptosis assay on an FACS system.

### Isolation and Identification of Astrocyte-Derived Exosome

The separation of exosomes from astrocytes was carried out as previously described (35). Briefly, supernatant collected from cultured astrocytes and human plasma was first centrifuged at 2 × 10^3^ g for 30 min, and then the supernatant was re-centrifuged at 1 × 10^5^ g for 45 min. The supernatant was filtered through a 0.45 μm filter to remove the large debris and dead cells. Small cell debris were removed by centrifugation at 1 × 10^6^ g for 70 min, and the supernatant was re-centrifuged at 1 × 10^6^ g for 70 min. The exosomes were cryopreserved at −80°C.

Particle size analysis of exosome samples were determined by using Particle Size Analyzer (NanoFCM N30E), 10 μl of samples were diluted to 30 μl. Exosome concentrations were determined using the BSA assay. After the instrument performance test with the standard sample was conducted, the exosome samples were loaded.

Transmission electron microscope (TEM) was used to observe the morphology of exosomes. A 10-μl sample was added to the copper net and incubated for 5 min at room temperature, and then the floating liquid was absorbed by the filter paper. The preparations so obtained were photographed under the transmission electron microscope (Hitachi, HT-7700).

### Fluorescence Labeling and Nano-Flow Cytometry of Exosomes

Samples of 20 μl exosome were diluted to 90 μl. Approximately 30 μl diluted exosomes were added to 20 μl fluorescein-labeled antibodies (CD9, CD63, IgG). The samples were mixed and incubated at 37°C for 30 min in the dark. The samples were added with 1 ml PBS, and then centrifuged at 4°C, 110,000*g* for 70 min. Then the supernatant was discarded, the samples were added with 1 ml PBS, and then re-centrifuged at 11,000*g* for 70 min. The supernatant was removed and 50 μl PBS was used to suspend samples. Exosomal proteins CD9 and CD63 expression were measured using Nano-Flow analysis (NanoFCM, Xiamen, China).

### Exosome Labeling and Uptake by BV2 Microglia Cell

By following the manufacturer’s instructions, PKH26 (Sigma Aldrich), a lipophilic dye, was used to label exosomes. Then the PKH26-labeled exosomes and BV2 microglial cells were co-cultured for 24 h. Subsequently, the cells were strained with 4′,6-diamidino 2-phenylindole (DAPI) and phalloidin (Sigma-Aldrich). Confocal laser scanning microscope (Olympus, Japan; FV1200) was used for recording and analysis.

### LncRNA and miRNA Analysis

The LncRNA and miRNA analysis was conducted by using Haplox (Jiang Xi, China). Exosomes were extracted by ultra-centrifugation. RNA purity was checked by using the NanoDrop™ One/OneC (Thermo Fisher Scientific, USA). RNA concentration was measured by employing Qubit^®^ RNA HS Assay Kit in Qubit^®^ 3.0 Flurometer (Life Technologies, USA). Next, about 3 µg RNA per sample was used for establishing the small RNA library. According to manufacturer’s instructions, sequence libraries were created using NEBNext^®^ Multiplex Small RNA Library Prep Set for Illumina^®^ (NEB, USA) and index codes were assigned to attribute sequences to each sample. The clustering of the index-coded samples was performed on a cBot Cluster Generation System using TruSeq PE Cluster Kit v3-cBot-HS (Illumia). After cluster generation, the prepared library was sequenced on an Illumina platform.

### Firefly Luciferase Reporter Assay

By querying the Starbase database, we found that INPP4 was the potential target gene of miR-137-5p, and miR-137-5p was a target of lncRNA GAS5. Luciferase reporter assay was carried out to verify these bioinformatic results. Commercial kit (Promega, USA; E1910) was used for fluorescence detection. The intensity of fluorescence was measured on a microplate reader (Perkin Elmer, USA, EnSight). All procedures were carried out in accordance with the manufacturer’s instructions.

In brief, BV2 microglial cells were seeded on a 24-well plate at a density of approximately 5 × 10^4^ cells/well for 24 h before transfection. Then, they were transfected with a plasmid containing the firefly luciferase gene, which has a complementary miR-137-5p binding site in its 3’ untranslated region (UTR) and was procured from GenePharma (Shanghai, China). Luciferase activity was expressed in relative light units (RLU).

### Statistical Analysis

Data were analyzed by using Prism 7 software (GraphPad, LaJolla, CA, USA). Categorical variables were compared by using the χ^2^ test or Fisher’s exact test. Continuous variables were compared by using the Student’s *t* test for variables that followed the normal distribution pattern. Otherwise, Mann–Whitney *U* test was utilized. The survival curves were analyzed using the Log-rank (Mantel–Cox) test. Data were expressed as mean ± SEM or median, percentage. The conventional two-sided tests were used in all analyses and the statistical significance level was set at 0.05.

## Results

### Exosomes From the Blood of CA/CPR Patients and Supernatant of OGD/R-Stimulated Primary Astrocyte

**Figure 1:**
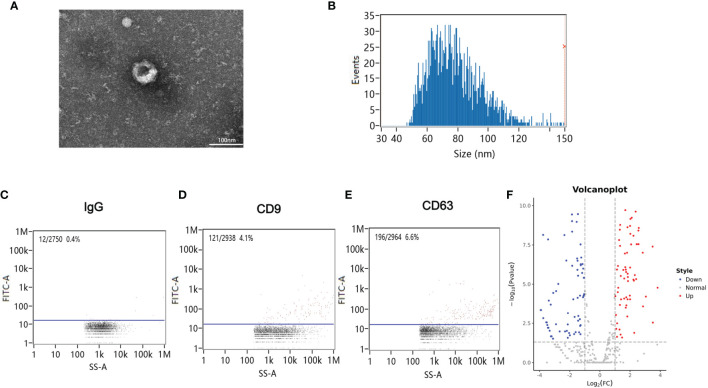
Exosomes in the plasma of cardiac arrest/cardiac pulmonary resuscitation (CA/CPR) patients. **(A)** A representative TEM image of purified exosomes from the CA/CPR patients. Scale bar 100 nm. **(B)** Particle size analysis of exosome samples detected by particle size analyzer. **(C–E)** Concentration of exosomes determined by nano-flowcytometry. **(F)** The miRNA distribution. The horizontal axis represents multiple miRNAs in the CA/CPR group *vs*. those in the healthy group, and the vertical axis represents the Log10 of the *p-*value.

**Figure 2:**
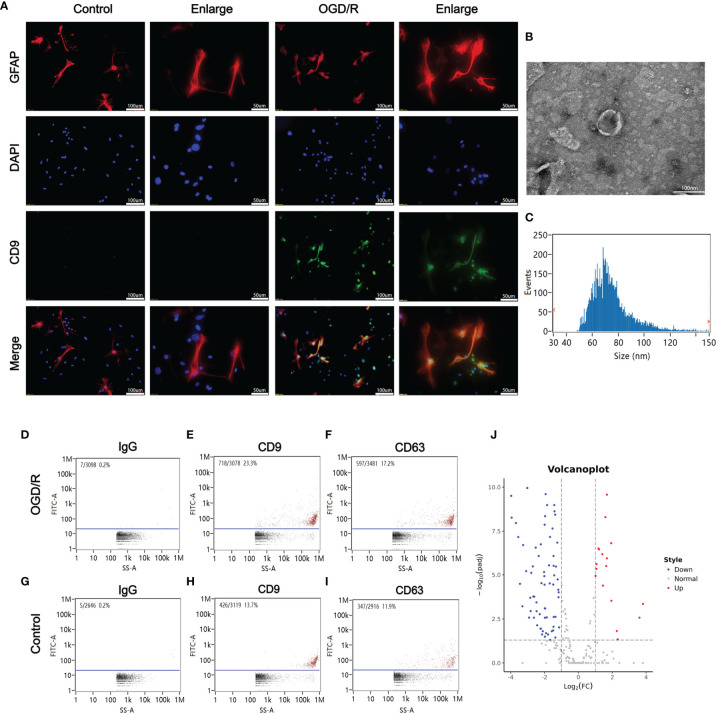
Oxygen glucose deprivation/reoxygenation (OGD/R) stimulated the release of astrocytes-derived exosomes. **(A)** Astrocytes-derived exosomes were significantly increased under oxygen glucose deprivation 4 h/reoxygenation 8 h (OGD 4 h/R 8 h). **(B)** A representative TEM image of purified exosomes of stimulated astrocytes after OGD/R. Scale bar 100 nm. **(C)** Particle size analysis of exosome samples detected by particle size analyzer. **(D–F)** The exosomes from the OGD/R-stimulated astrocytes were identified by CD9 and CD63 using nano-flowcytometry. **(G–I)** The exosomes from the control astrocytes were identified by CD9 and CD63 using nano-flowcytometry. **(J)** The miRNA distribution. The horizontal axis represents multiple miRNAs in the OGD/R group *vs*. those in the control group, and the vertical axis represents the Log10 of the *p-*value.

Blood samples from patients were collected 12 h after CA/CPR. **Supplementary Table 1** details the information of enrolled patients suffering from CA/CPR. **Figure 1** shows images of transmission electron microscopy, particle sizes, and concentrations of purified exosomes from the cardiac arrest patients. Our results demonstrated, for the first time, that CA/CPR patients released exosomes into the blood under the insult of acute systemic ischemia–reperfusion injury. **Figure 2** shows that OGD/R-stimulated primary astrocytes synthesized and released exosomes.

### miRNA Expression Profiles of Exosomes in the Blood From CA/CPR Patients and the Supernatant of OGD/R-Stimulated Astrocytes

To investigate the mechanisms of CA/CPR-associated multiply organ dysfunction, we employed the miRNA assay to explore the miRNA expression profiles of exosomes in peripheral blood from CA/CPR patients and the supernatant of OGD/R-stimulated astrocytes.

We assessed total RNA amount (CA patients ≥0.47 μg; astrocyte ≥0.32 μg), the sample concentration (CA patients ≥3.85 ng/μl; astrocyte ≥3.73ng/μl), and the purity of samples.

(CA patients, OD260/280 ≥1.28; astrocyte, OD260/280 ≥1.27). After high-throughput RNA sequencing and cleaning of low-quality sequences of the original data, we finally obtained 26.53 M reads and 1.33 G bases in the CA/CPR patients, 25.37 M reads and 1.26 G bases in the sham controls. Besides, 50.66 M clean reads and 2.53 G clean bases were obtained in astrocytes, containing 24.69 M reads and 1.23 G bases in the injury models, 25.97 M reads and 1.30 G bases in the sham controls. The effective base ratio was greater than 95%. The average guanine–cytosine content ranged between 54 and 55%.

Altogether, 22,219 protein-encoding transcripts and 31,811,734 small RNAs in CA patients were analyzed thoroughly. A total of 11,037 protein-encoding transcripts and 18,653,878 small RNAs in astrocytes were identified and subsequently analyzed in-depth. A total of 706 miRNAs in CA/CPR patients and 366 miRNAs in OGD/R-stimulated astrocytes were significantly altered compared to healthy subjects or sham controls, respectively. A total of 5,231 lncRNAs in CA/CPR patients and 1,403 lncRNAs in OGD/R-stimulated astrocytes were significantly changed in comparison with healthy subjects or sham controls, respectively. In CA patients, 842 mRNAs and 1,071 lncRNAs were upregulated, while 3,351 mRNAs and 4,161 lncRNAs were downregulated. In OGD/R-stimulated astrocytes, 695 mRNAs and 633 lncRNAs were upregulated, while 1,316 mRNAs and 770 lncRNAs were downregulated.

We then focused on small RNAs that were changed significantly in both CA/CPR patients and OGD/R injury cell model, in order to allow for research from the patient bed to lab bench. **Table 1** displays the top 40 most significantly regulated miRNAs (the 20 were upregulated and 20 downregulated) as revealed by differential expression analysis. **Figures 1F** and **2J** (volcano plot visualization) show the dramatically different expression levels of miRNAs in the CA/CPR patients and the supernatant of OGD/R-stimulated astrocytes, respectively.

**Table 1:** The most significantly regulated 40 miRNAs (the top 20 upregulated and the top 20 downregulated) by differential expression analysis which were extracted from exosomes of CA/CPR patients’ blood and OGD/R-stimulated astrocytes.

AccID	log2FC	*P*-value	FDR	Regulation
miR-138-5p	−3.491669477	2.05E−33	2.12E−31	down
miR-25-5p	−3.868073493	1.73E−24	1.23E−22	down
miR-92b-3p	−1.707369436	6.43E−16	2.45E−14	down
miR-192-5p	−1.710462151	1.04E−15	2.70E−14	down
miR-122-5p	−2.26738957	1.24E−15	4.57E−14	down
miR-122b-3p	−2.28149715	1.24E−15	4.57E−14	down
miR-137-5p	−10.69162188	1.99E−14	6.71E−13	down
miR-148a-5p	−4.706870352	5.57E−14	1.20E−12	down
miR-3960	−10.64584633	5.42E−14	1.62E−12	down
miR-877-5p	−2.410665284	3.94E−12	9.44E−11	down
miR-92b-5p	−3.023633383	4.78E−12	1.12E−10	down
miR-340-3p	−10.39226289	5.65E−12	1.29E−10	down
miR-744-5p	−1.848636606	5.65E−11	9.84E−10	down
miR-323b-3p	−8.970071701	1.02E−10	1.71E−09	down
miR-342-3p	−1.413307616	1.20E−10	2.36E−09	down
miR-1307-5p	−4.284191234	1.49E−10	2.46E−09	down
miR-4785	−1.8411616	2.87E−10	4.55E−09	down
miR-331-3p	−10.08439616	4.27E−10	7.45E−09	down
miR-29c-3p	−2.357009139	4.98E−10	7.59E−09	down
miR-125b-2-3p	−1.995632068	8.14E−10	1.36E−08	down
miR-6748-5p	11.89794699	2.33E−54	2.18E−51	up
miR-6131	3.464785036	5.27E−44	2.11E−41	up
miR-195-5p	2.85859883	4.80E−28	5.86E−26	up
miR-455-5p	10.33309058	6.09E−25	6.10E−23	up
miR-1469	6.79938632	9.01E−25	8.42E−23	up
miR-564	7.638856409	4.37E−24	3.83E−22	up
miR-3648	10.08176483	1.36E−21	8.27E−20	up
miR-4476	10.08176483	1.36E−21	8.27E−20	up
miR-3661	10.03526589	7.44E−21	4.26E−19	up
miR-4299	6.497088014	7.44E−21	4.26E−19	up
miR-3916	9.937515046	1.01E−19	4.73E−18	up
miR-1246	2.097937187	1.10E−19	5.07E−18	up
miR-572	9.748687181	9.21E−18	3.11E−16	up
miR-1343-5p	9.65952688	9.51E−17	2.90E−15	up
miR-19b-3p	2.661039351	2.97E−16	8.50E−15	up
novel-24	9.462751066	7.36E−15	1.76E−13	up
miR-20a-5p	2.71507084	7.61E−15	1.81E−13	up
miR-214-3p	1.802909007	4.58E−14	1.45E−12	up
miR-375-3p	1.832771029	3.15E−13	9.16E−12	up
miR-212-5p	10.57256259	3.84E−13	1.08E−11	up

### Construction of lncRNA/miRNA/mRNA Regulatory Network

**Figure 3:**
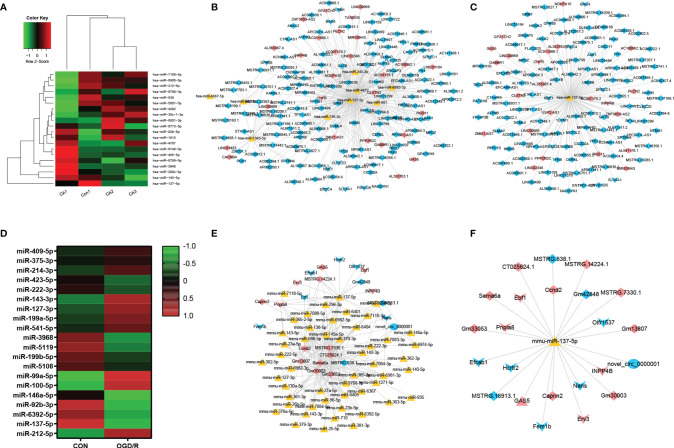
LncRNA/miRNA/mRNA axes were involved in the pathogenesis of acute ischemia/reperfusion injury. **(A)** The miRNA components of the exosomes were studied by microRNA microarray analysis. The heat map shows the 20 most significantly regulated miRNAs from CA/CPR patients. Con, exosomes from healthy subjects; CA, exosomes from CA/CPR patients. **(B)** The differential miRNA bound with lncRNAs–mRNAs were predicted by Miranda 3.3 and rnahybrid 2.1.2 software. Red and blue represent upregulation and downregulation respectively; triangle represents miRNA, arrow is mRNA and diamond denotes lncRNA. **(C)** LncRNA/miRNA/mRNA network with hss-miR-137-5p as the core. **(D)** The miRNA components of the exosomes were studied by microRNA microarray analysis. The heat map shows the 20 most significantly regulated miRNAs from the OGD/R-stimulated astrocytes. Con, exosomes secreted by astrocytes under physiological conditions; OGD/R, exosomes secreted from the OGD/R-stimulated astrocytes. **(E)** The differential miRNA bound with lncRNAs–mRNAs and ceRNAs were predicted by Miranda 3.3 and rnahybrid 2.1.2 software. Red and blue represent upregulation and downregulation respectively, triangle represents miRNA, arrow is mRNA, diamond denotes lncRNA and circle indicates ceRNA. **(F)** LncRNA/miRNA/mRNA network with mmu-miR-137-5p as the core.

A total of 56,818 lncRNA/miRNA/mRNA target pairs were identified in CA/CPR patients, namely, 164 lncRNAs, 620 miRNAs, and 16 mRNAs. Similarly, a total of 1,387 lncRNA/miRNA/mRNA target pairs were identified in the supernatant of OGD/R-stimulated astrocytes, namely, 13 lncRNAs, 290 miRNAs, 10 mRNAs, and 1 ceRNA. **Figures 3A, D** respectively demonstrate miRNA components of the exosomes from CA/CPR patients or the supernatant of OGD/R-stimulated astrocytes. **Figures 3B**, **E** respectively show the co-expression network analysis of differentially-expressed lncRNA/miRNA/mRNA from peripheral blood of CA/CPR patients or the supernatant of OGD/R-stimulated astrocytes.

On the basis of the microarray analysis, previous studies (36–39) and database search from the bioinformatic website (http://starbase.sysu.edu.cn), we selected miR-137 as our research target. Our analysis showed that miR-137 was among the top 10 miRNAs that experienced most changes in blood of CA/CPR patients and the supernatant of OGD/R-stimulated astrocytes. **Figures 3C, F** respectively exhibit the network of lncRNA/miRNA/mRNA, with miR-137 as the core miRNA from peripheral blood of CA/CPR patients or the supernatant of OGD/R-stimulated astrocytes.

**Figure 4:**
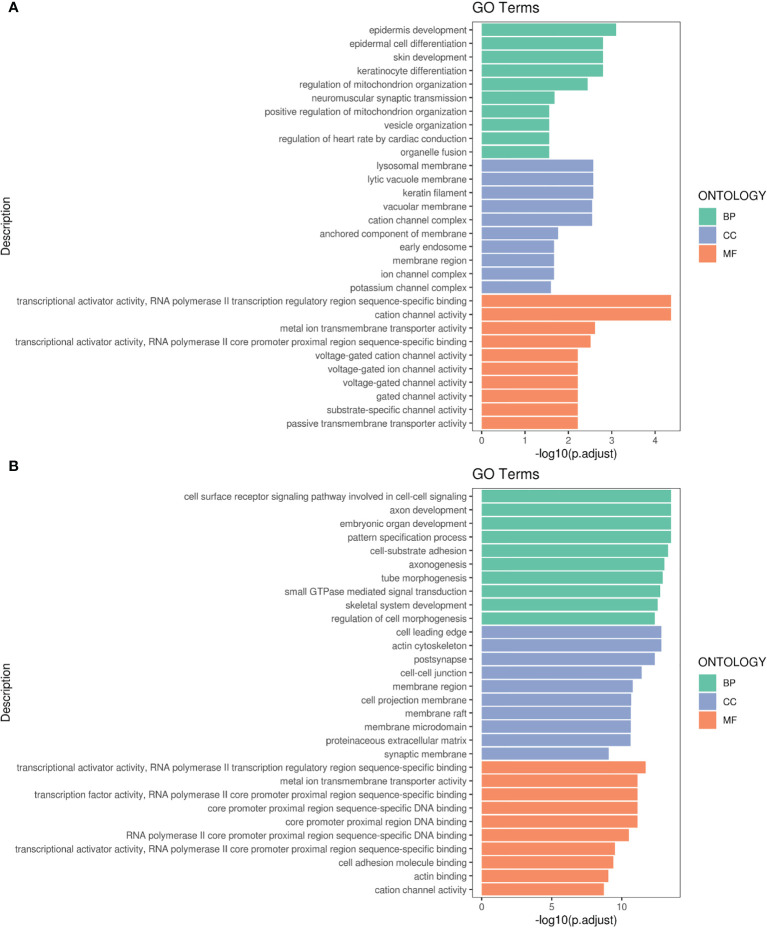
Gene Ontology (GO) pathway enrichment analysis of differentially-expressed RNA. **(A)** The top 10 of the reads count in CA/CPR patients. **(B)** The top 10 of the reads count in OGD/R-stimulated astrocytes. BP, biological processes; CC, the enrichment of cellular components; MF, the enrichment of molecular functions.

**Figures 4A, B** respectively display the results of GO pathway enrichment analysis of differentially-expressed miRNAs from peripheral blood of CA/CPR patients or the supernatant of OGD/R-stimulated astrocytes. The GO analysis revealed that these lncRNA/miRNA/mRNA axes were involved in the regulation of cytokine–cytokine receptor interaction, cholinergic synapses, structural and functional abnormalities of mitochondrial respiratory chain, mitophagy, B cell apoptosis, epilepsy, modulation of the L-type calcium channel, ubiquitination, sumolyation, the Jak-STAT signaling pathway, the PI3K-Akt signaling pathway, among others.

However, all results inferred from bioinformatic analysis should be verified by the clinical studies, *in vitro* and *in vivo*. In the following research, we investigated how lncRNA regulates mRNA levels through miR-137 and what role it plays in the pathological mechanism of multiple organ dysfunction after CA/CPR.

### LncRNA GAS5 Negatively Regulates miR-137 Expression

**Figure 5:**
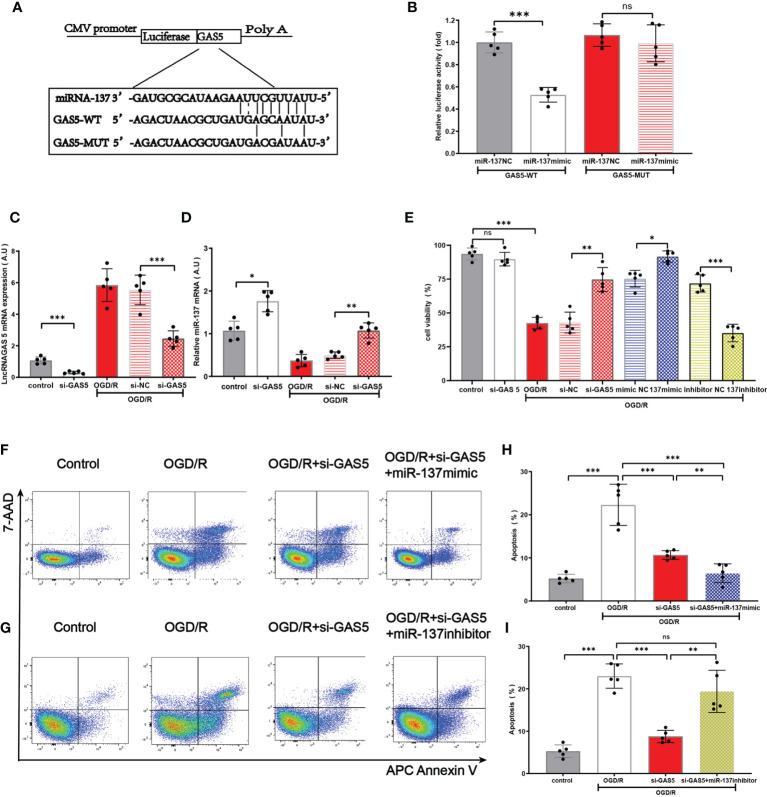
LncRNA GAS5 targets miR-137 and negatively regulates the expression of miR-137. **(A)** The putative miR-137 bound sequence of the wild type (WT) and mutant (MUT) sequence of LncRNA GAS5 from the bioinformatic website (http://starbase.sysu.edu.cn). **(B)** Luciferase reporter assay was conducted to verify these bioinformatic results. GAS5-WT, LncRNAGAS5-wild type; GAS5-MUT, mutant LncRNA GAS5; miR-137 NC, miRNA 137 negative control. **(C, D)** Quantitative PCR was used to detect the expression of miR-137 and LncRNAGAS5 in microglia after transfection with GAS5 siRNA and exposure to OGD/R damage. **(E)** Cell viability was detected in different groups by MTT assay. **(F, G)** Representative flow cytometrical data of Annexin V expression in microglia cells exposed to OGD/R in different groups. **(H, I)** Bar graph displays the frequency of Annexin V^+^ microglial cells exposed to OGD/R in different groups. Data were presented as mean ± SEM, *p <0.05, **p <0.01, ***p <0.001, and ns indicates no significant difference, (n = 5).

On the basis of previous research (12, 40, 41) and database search in the bioinformatic website (http://starbase.sysu.edu.cn), we found that lncRNAGAS5 contained one conserved target site of miR-137 (**Figure 5A**). Further dual-luciferase reporter assay revealed that miR-137 mimic remarkably inhibited the luciferase activity of lncRNAGAS5-WT rather than lncRNAGAS5-MUT (**Figure 5B**).

Under the insult of OGD/R, the expression of lncRNAGAS5 in astrocyte was increased, and the expression of miR-137 in astrocyte was decreased. Notably, silencing lncRNAGAS5 in OGD/R-injured astrocytes significantly upregulated miR-137 expression, compared with the controls (**Figures 5C, D**). **Figure 5E** shows that silencing lncRNAGAS5 significantly enhanced cell viability and mitigated OGD/R-induced apoptosis. Furthermore, in miR-137 mimic group, the survival percentage was higher while apoptosis percentage was lower in OGD/R-injured astrocyte as compared to transfection with si-GAS5 alone. Nonetheless, miR-137 inhibitor virtually abolished this beneficial effects of si-GAS5 on survival and apoptosis under OGD/R injury (**Figures 5F**–**I**).

Collectively, lncRNAGAS5 negatively regulated miR-137 expression by serving as a molecular sponge for miR-137. Silencing lncRNAGAS5 elevated miR-137 expression and ameliorated OGD/R-induced injury in astrocyte.

### miR-137 Targets Inositol Polyphosphate 4-Phosphatases (INPP4B) and Inhibits its Expression

**Figure 6:**
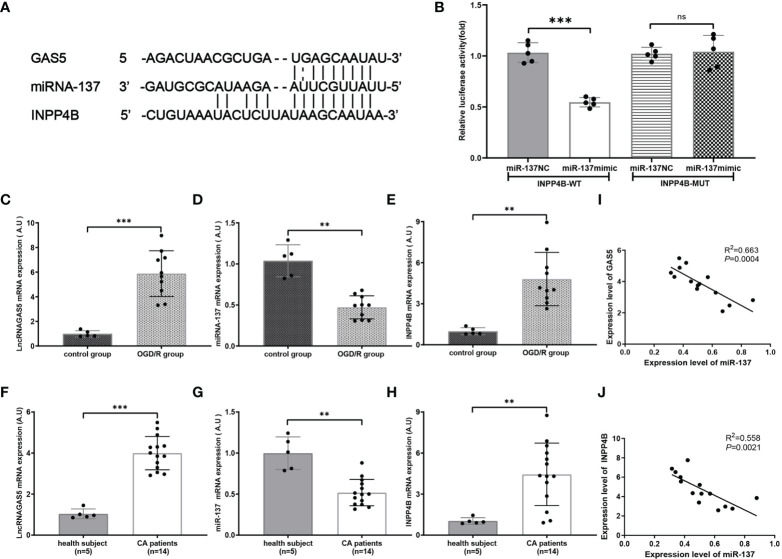
miR-137 targets inositol polyphosphate 4-phosphatases (INPP4B) and inhibits its expression. **(A)** The putative sites where lncRNA GAS5 binds with miR-137, and the sites where miR-137 binds with NIPP4B (http://starbase.sysu.edu.cn). **(B)** Luciferase reporter assay was carried out to verify these biostatistic results. INPP4B-WT, INPP4B-wild type; INPP4B-MUT, mutant INPP4B; miR-137 NC, miRNA 137 negative control. **(C–E)** Quantitative RT-PCR was performed to detect the expression of lncRNA GAS5, miR-137, and INPPB from CA/CPR patients, respectively. **(F–H)** Quantitative PCR was conducted to detect the expression of lncRNA GAS5, miR-137 and INPP4B in the microglia after OGD/R treatment, respectively. **(I, J)** Pearson’s correlation analysis of the relationship among lncRNA GAS5, miR-137 and INPP4B in CA/CPR patients. Data were expressed as mean ± SEM, *p <0.05, **p <0.01, ***p <0.001, and ns indicates no significant difference, (n = 5).

By searching two major miR target prediction databases, miRanda (42) and Targetscan (43), our bioinformatic analysis (**Figure 6A**) indicated that miR-137 acted as a potential regulator of INPP4B. Luciferase activity assay confirmed that INPP4B was a direct target of miR-137 (**Figure 6B**). Hence, we further examined the relationship among lncRNA GAS5/miR-137/INPP4B both *in vivo* and *in vitro*.

We determined the mRNA expression of lncRNA GAS5, miR-137, and INPP4B by using qRT-PCR in the OGD/R group. As shown in **Figures 6C**–**E**, under the stimulation of OGD/R, lncRNA GAS5 and INPP4B were upregulated and miR-137 was downregulated.

Most intriguingly, qRT-PCR showed that lncRNA GAS5 and INPP4 were upregulated and miR-137 were downregulated in blood sample from CA/CPR patients as compared to the healthy subjects (**Figures 6F**–**H**). Comparable changes were observed in the OGD/R-stimulated astrocytes. Additionally, our results revealed that the expression level of lncRNA GAS5 was negatively correlated with miR-137 expression, and the miR-137 expression was also negatively correlated with INPP4 mRNA in the blood sample from CA/CPR patients (**Figures 6I, J**).

### LncRNA GAS5/miR-137 Have Obvious Effect on the Modulation of Apoptosis and Inflammatory Phenotype Transformation of Microglia After OGD/R Injury

**Figure 7:**
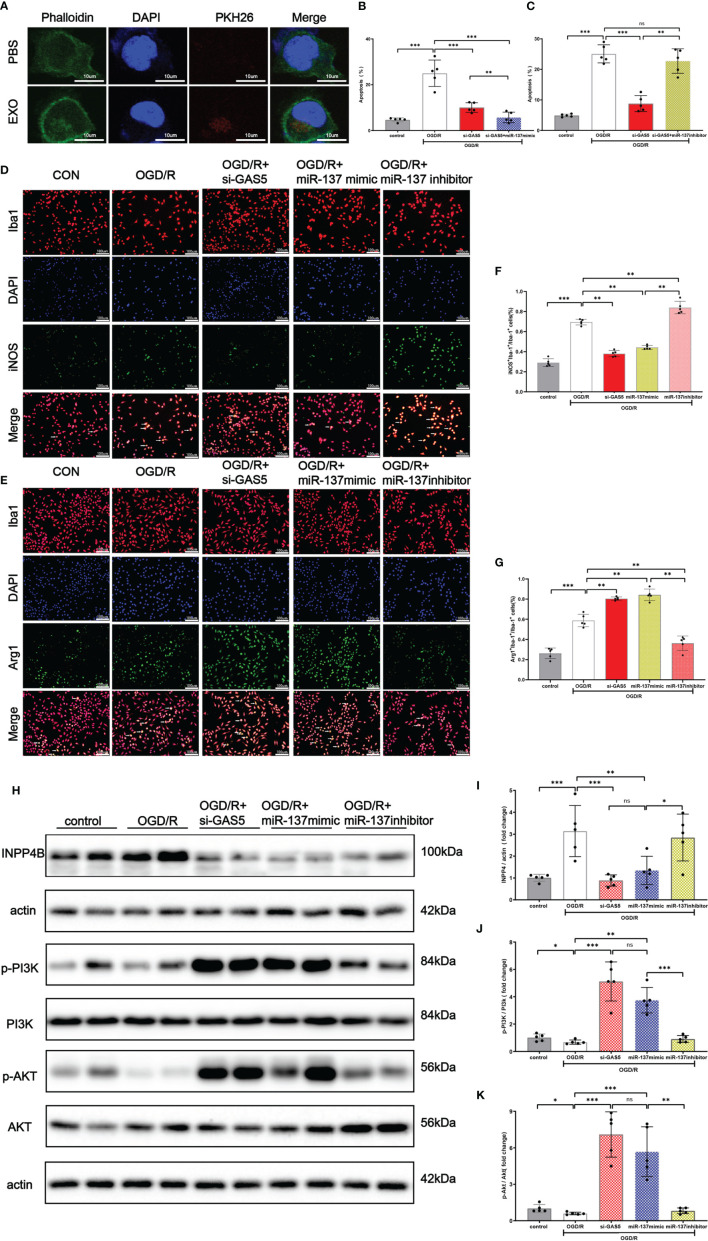
Exosomes were uptaken by microglia. Upregulated miR-137 expression mitigated apoptosis and promoted microglial transformation to anti-inflammatory phenotype after OGD/R injury. **(A)** An exosome taken up by a microglion. A representative immunofluorescence image of a primary microglion showing the nucleus (DAPI, blue), an exosome (PKH26, red) and F-actin (green). **(B)** Bar graph displays the apoptosis rate of microglial cells transfected with si-GAS5 alone or together with miR-137 mimic exposed to OGD/R. **(C)** Bar graph displays the apoptosis rate of microglial cells transfected with si-GAS5 alone or together with miR-137 inhibitor exposed to OGD/R. **(D, E)** Representative fluorescence confocal microscopic images show that Iba-1, iNOS (M1 marker of microglia), and Arg-1 (M2 marker of microglia) expression after OGD/R treatment in different groups. Bar = 50 μm. **(F)** The percentages of Arg-1^+^Iba-1^+^ cells in total Iba-1^+^ cells in different groups. **(G)** The percentages of iNOS^+^Iba-1^+^ cells in total Iba-1^+^ cells in different groups. **(H–K)** Protein expression of INPP4B, PI3K, AKT, and their phosphorylation in the microglia exposed to OGD/R and the representative dot plots. (The data are presented as mean ± SEM, *p <0.05, **p <0.01, ***p <0.001, and ns indicates no significant difference, n = 5).

As shown in **Figure 7A**, exosomes, stained with PKH26, were taken up by microglia. Flow cytometry results (**Figures 7B, C**) revealed that miR-137 mimic or silencing LncRNA GAS5 significantly mitigated apoptosis when microglia suffered from OGD/R injury, whereas miR-137 inhibitor significantly promote microglia apoptosis. Moreover, our results showed that upon the stimulation of OGD/R, microglia transformed to pro-inflammatory phenotype, known as M1 microglia, and miR-137 mimic or silencing LncRNA GAS5 significantly promoted the transformation to microglia M2 (anti-inflammatory) phenotype, and miR-137 inhibitor significantly promoted transformation to microglia M1 (pro-inflammatory) phenotype (**Figures 7D**–**G**).

Western blot analysis demonstrated (**Figures 7H, J, K**) that INPP4B protein expression was increased, with accompanied decrease in PI3K and Akt phosphorylation in the OGD/R group. When LncRNA GAS5 was silenced or miR-137 was over-expressed, no significant change was observed in PI3K and Akt protein expression while their phosphorylation was increased (p <0.05). Notably, miR-137 inhibitor practically counteracted PI3K and Akt phosphorylation, which almost reached the expression level of OGD/R group (**Figures 7J, K**).These results suggested that knockout of LncRNA GAS5 or over-expression of miR-137 activated the PI3K/Akt phosphorylation, while inhibition of miR-137 reversed beneficial effect resulting from the silencing of LncRNA GAS5 under OGD/R insults.

Moreover, **Figures 7H** **, I** show that the INPP4B protein expression was remarkably elevated in the OGD/R group, while its expression was inhibited in the OGD + si LncRNA GAS 5 group or OGD + miR-137 mimic group. The findings were consistent with results of the mRNA expression of LncRNA GAS5, miR-137, and INPP4B shown by qRT-PCR when the microglia were exposed to OGD/R. Taken together, silencing LncRNA GAS5 suppressed INPP4B expression, and miR-137 negatively regulated their expression.

### Over-Expression of miR-137 Attenuated Microglial Apoptosis and Neuro-Inflammation in Murine CA/CPR Model

**Figure 8:**
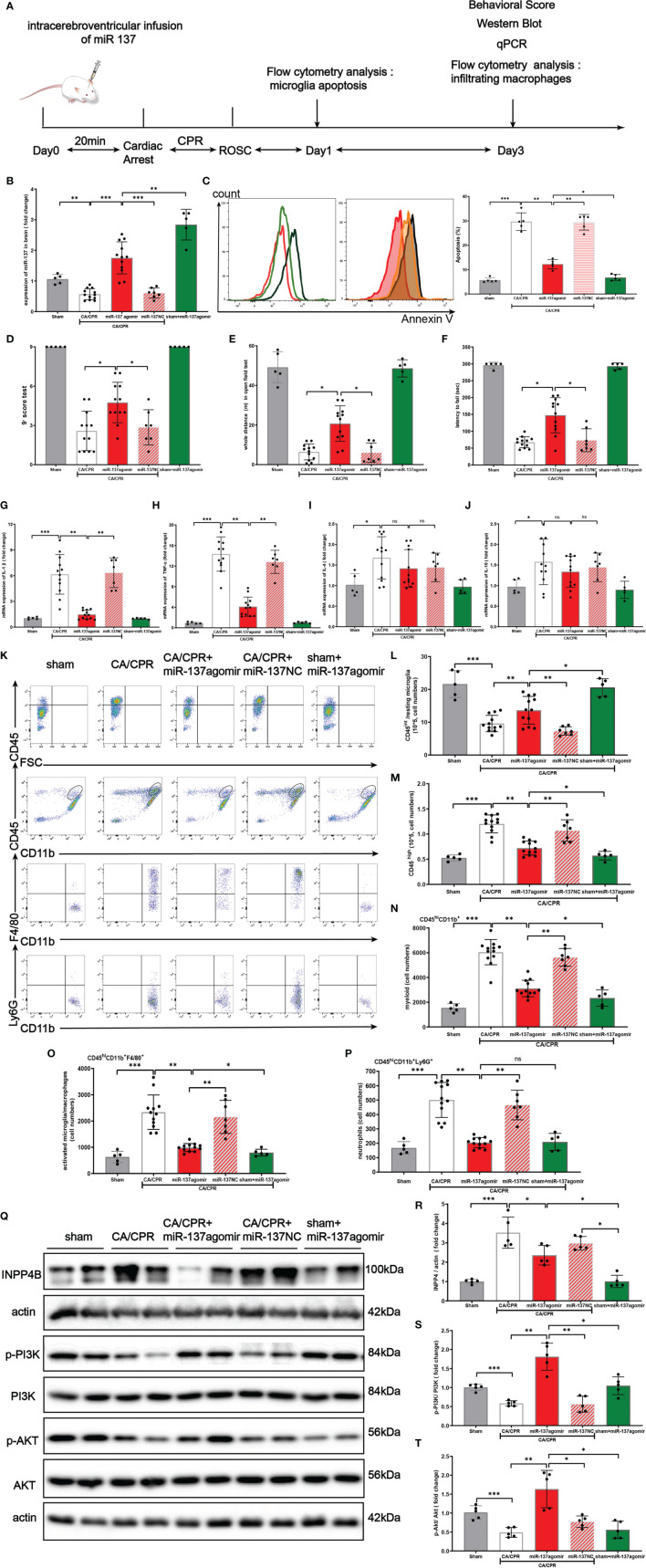
Over-expression of miR-137 attenuated microglial apoptosis and neuro-inflammation in cardiac arrest/cardiac pulmonary resuscitation mice model. **(A)** Schematic diagram of the experimental design. **(B)** The expression of miR-137 miRNA level in brain tissues in different groups was detected by Northern blot. **(C)** Representative image of fluorescence-activated cell sorter (FACS) staining of Annexin V expression in microglial cells on day 1 after CA/CPR in different groups. Sham controls (red), CA/CPR group (black), CA/CPR with miR-137 NC (green hollow histogram) and CA/CPR with miR-137 mimic (orange filled histogram). Bar graph displays the frequency of Annexin V^+^ cells on day 1 after CA/CPR in different groups (n = 5 per group). Data are expressed as means ± SEM. *p <0.05, **p <0.01, Student’s *t*-test. **(D–F)** Neurologic scoring and behavioral tests were performed at the indicated time points (n = 5–12/group). **(G–J)** On day 3 after CA, the mRNA expression of IL-1β, TNFα, IL-4, and IL-10 in brain were detected by qPCR in different groups. **(K–P)** On day 3 after CA, the number of resting microglial cells (CD45^int^/CD45^int^ CD11b^+^), activated microglia/macrophages (CD45^hi^CD11b^+^F4/80^+^), and neutrophils (CD45^hi^CD11b^+^Ly6G^+^) were quantified and the representative dot plots are shown. Data are presented as mean ± SEM. *p <0.05, **p <0.01, n = 5–12/group. **(Q–T)** Protein expression of INPP4B, PI3K and AKT in brain tissues was detected by Western blotting (data are presented as mean ± SD, *p <0.05, **p <0.01, ***p <0.001, and ns indicates no significant difference, n = 5).

**Figure 8A** shows the schematic diagram of the experimental design. In brief, C57BL/6 mice were intracerebro-ventricularly infused miR-137 agomir or NC (negative control) with 20 min before CA/CPR surgery. The mice were randomly divided into 5 groups by using the online tool Quickcalcs: i.e., sham surgery group (only subjected to anesthetization and tracheal intubation), CA/CPR group, CA/CPR + miR-137 agomir group, sham + miR-137 NC group, and CA/CPR + miR-137 NC group, respectively. In the preliminary experiment, we determined miR-137 mRNA levels in all groups by Northern blotting (**Figure 8B**).

In the preliminary experiment, we set up a group of CA/CPR + miR-137 antagomir. Despite great effort, no animals survived till the final analysis and they died within 18 h after CA/CPR surgery (**Supplementary Chart 1**).

On post-CA day 1, as shown in **Figure 8C**, CA/CPR significantly induced apoptosis of microglia and miR-137 agomir reduced apoptosis while miR-137 NC had no effect on apoptosis. Consistently, flow cytometry showed that the number of resting microglial cells (CD45^int^/CD45^int^ CD11b^+^) conspicuously dropped, while miR-137 agomir group partially restored the number of resting microglial cells on post-CA day 3 (**Figure 8L**).

On post-CA day 3, compared to CA/CPR mice, miR-137 agomir group performed significantly better on behavioral tests, in terms of neurologic scores (**Figure 8D**), distance of open field test (**Figure 8E**) and rotarod time (**Figure 8F**), while miR-137 NC group showed no improvement in performance on post-CA behavioral tests. Nevertheless, no significant difference was found in the overall survival rate between the CA/CPR group and miR-137 agomir group (46.2% *vs.* 75%, *p* = 0.1269, **Supplementary Figure 1**), whereas there existed a significant difference in survival rate between the CA/CPR group and the sham group (46.2% *vs.* 100%, *p* = 0.0488, **Supplementary Figure 1**).

On post-CA day 3, brain samples were collected for PCR and flow cytometry. Compared to the sham group, mRNA levels of IL-1β, TNFα, IL-4, and IL-10 were significantly increased in CA/CPR mice. Additionally, the increases in IL-1β and TNFα were largely suppressed by miR-137 agomir, compared to that of CA/CPR group. Neither IL-4 nor IL-10 was suppressed by miR-137 agomir (**Figures 8G**–**J**).

We further investigated the effect of miR-137 agomir on activated microglia/macrophages (**Figures 8K**–**P**). We found that the number of CD45^hi^ CD11b^+^ was significantly increased in the brain on day 3 post CA, and that most of these cells were activated microglia/macrophages and neutrophils, identified as the CD45^hi^ CD11b^+^ F4/80^+^, CD45^hi^CD11b^+^ Ly6G ^+^ subpopulations, respectively. Notably, over-expression of miR-137 could evidently reduce the number of CD45^hi^CD11b^+^F4/80^+^and CD45^hi^CD11b^+^ Ly6G ^+^ subpopulations, and miR-137 NC did not have such effect.

Taken together, the levels of cytokines and counts of resting microglia, activated microglia/macrophages in the post-CA brain revealed that over-expression of miR-137 could potentially inhibit apoptosis and neuro-inflammatory response in the post-CA brain.

In **Figures 8Q**–**T**, Western blotting showed that CA/CPR surgery increased INPP4B protein expression, inhibited phosphorylation of PI3K/Akt signaling pathway. Over-expression of miR-137 significantly activated the PI3K/Akt signaling pathway, which is likely to be the mechanism by which microglial apoptosis and neuro-inflammation are inhibited during PCAS.

## Discussion

Neurocytes, astrocytes, and microglia are the most abundant cell types in the central nervous system (CNS). Under the physiological conditions, astrocytes and microglia play a crucial role in maintaining immune balance, microenvironment stability, and neural circuit function (19, 44). When the brain sustains injury, astrocytes are activated and they send negative feedback to activate microglia, thereby influencing CNS immunity and inflammation (19, 44, 45). Recently, the role of non-protein coding RNAs in the immune responses has been a subject of active studies. In this study, we explored the roles of lncRNAs/miRNAs/mRNAs in the regulation of post-CA/CPR neuro-inflammation.

In this study, we found that the brain-enriched miR-137 was a hypoxia responsive miRNA in the miRNA profile. We confirmed that astrocyte-derived exosomes were taken up by microglia under the OGD/R insult. Our miRNA microarray analysis showed that miR-137 was among the top 10 upregulated miRNA in the exosome synthesis and released by OGD/R-stimulated astrocytes. Endogenous miR-137 in the OGD/R-stimulated group dropped significantly compared with control group as revealed by gene differential expression comparison and GO enrichment analysis. Downregulated miR-137 was significantly detected in blood samples from CA/CPR patients compared to the healthy subjects.

Downregulated miR-137 in response to hypoxia *in vitro* and *in vivo* was consistent with previous studies (36, 37, 39–41). miR-137 was also downregulated in the serum of patients with Alzheimer’s disease (46). Li et al. showed that the miR-137 was remarkably inhibited in mouse brains or cells under hypoxic conditions (37).

Notably, we found that the microglial apoptosis was increased remarkably, and microglia were more inclined to transform to M1 phenotype by downregulating the miR-137 expression in the OGD/R cell experiments. On the contrary, the apoptosis of microglia was largely suppressed and the microglia tended to transform to M2 phenotype by upregulating the miR-137 expression. We further revealed that upregulated miR-137 exerted anti-apoptotic and anti-inflammatory effects in CA/CPR mice model, and had a beneficial effect on the behavioral and neurological scores three days after CA/CPR surgery, although no significant difference was found in the overall survival rate between the CA/CPR group and miR-137 agomir groups (**Supplementary Figure 1**).

By bioinformatic analysis (http://starbase.sysu.edu.cn), the upstream and downstream target genes of miR-137 were obtained. On the basis of search results of Gene Ontology (GO) databases, we speculated that miR-137 could negatively regulate the expression of inositol polyphosphate 4-phosphatases (INPP4) in microglia.

Mounting evidence has demonstrated that the ubiquitously expressed phosphoinositide 3-kinase (PI3K) family of lipid kinases controls a wide array of cellular functions, namely, cell survival, proliferation, metabolism, and migration (47). A number of PIP3-binding proteins also bind to PI (3, 4)P2, such as the protein kinase Akt/PKB, the most studied effector of PI3K signaling. PI3K/Akt are well-established players in cell survival and proliferation, and inositol polyphosphate 4-phosphatases (INPP4A and INPP4B) negatively regulate PI3K by selectively degrading PI (3, 4) P2, which is one of PI3K components (47, 48).

We looked into the relationship among the miR-137, INPP4B, and PI3K/Akt pathway in OGD/R cell injury model and CA/CPR animal model, respectively. The results from the OGD/R cell model showed that upregulation of miR-137 could significantly inhibit INPP4 expression and promote the activation of PI3K/Akt pathway, while downregulation of miR-137 could remarkably increase INPP4 expression and suppress the activation of PI3K/Akt pathway. Moreover, in CA/CPR animal model, we observed that over-expression of miR-137 could inhibit expression of INPP4B. Although no significant change in the INPP4B expression was found in the CA/CPR + miR-137 agomir group compared with miR-137 NC group (*p* = 0.057) and its PI3K/Akt pathway was significantly phosphorylated in the miR-137 agomir group.

The aforementioned results might be ascribed to following factors: The microenvironment of animal experiments is much more complicated than that of cell experiments. As other active compounds might also be involved in the recipient cells, miRNA in exosomes is likely to be only a part of entire maze. For example, miR-210 and miR-21 are well-established hypoxia-responsive microRNAs, which are distinctly upregulated in mouse brain and cells subjected to hypoxia stress (49). It is understandable that miR-137/INPP4B/PI3K/Akt pathway is one axis of a huge network. In other words, the upregulated miR-137 promoted the phosphorylation of PI3K/Akt pathway, and INPP4B may not be the unique link bridging both of them. This also stresses a notion that all the results of bioinformatic analysis must be verified by both cell and animal experiments (33, 50).

Our study also focused on lncRNAs that compete with target miRNA genes by binding to the shared miRNA sequence, thereby reducing the degradation of the target mRNA. Small RNA microarray analysis of the exosome showed that miR-137 was downregulated and lncRNA GAS5 was apparently upregulated. Bioinformatic analysis revealed that lncRNA GAS5 contained one conserved target site of miR-137. Previous research demonstrated that the Growth Arrest-Specific 5 (lncRNA GAS5) negatively regulated cell survival and was upregulated in the neurons that responded to hypoxia (40). Chen et al. showed that LncRNA GAS5 was apparently increased, and miR-137 expression was decreased in mice subjected to MCAO surgery and in OGD-injured murine neurons. LncRNA GAS5 might aggravate the progression of ischemic stroke through miR-137 mediated Notch1 signaling pathway. Knockdown of GAS5 could attenuate neuronal injury induced by OGD/R (12, 40, 41).

Our *in vitro* results indicated that lncRNA GAS5 might serve as a molecular sponge for miR-137 and negatively regulated its expression. The dual-luciferase reporter assay exhibited that miR-137 mimic but not NC-mimic distinctly suppressed the luciferase activity of lncRNA GAS5-WT. Silencing lncRNA GAS5 in the astrocyte following OGD/R remarkably upregulated miR-137 expression. Furthermore, in OGD/R-stimulated astrocyte–microglia cell culture, silencing lncRNA GAS5 decreased apoptosis and pro-inflammatory factors in the OGD/R stimulated group, while downregulating miR-137 could strikingly increase apoptosis and pro-inflammatory factors. In addition, miR-137 mimic could reverse the effect of miR-137 inhibitor on microglial cells, showing it could reduce apoptosis and promote the transformation of microglia to M2 phenotype.

To the best of our knowledge, we demonstrated, for the first time, that small RNA results of blood samples from CA/CPR patients were consistent with findings of OGD/R cells. We found that (1) lncRNA GAS5 was highly expressed in the blood of CA/CPR patients, brain tissue of CA/CPR mice and OGD/R stimulated astrocytes (2). The expression level of lncRNA GAS5 was negatively correlated with miR-137 expression, and the miR-137 expression was negatively correlated with INPP4 mRNA level in blood samples from CA/CPR patients (3). lncRNA GAS5 and INPP4B were upregulated while miR-137 was downregulated in blood samples from CA/CPR patients when compared to the healthy subjects. The similar trends were observed in the OGD/R-stimulated astrocyte–microglia cell culture as compared to the control group.

Taken together, our results demonstrated that lncRNA GAS5/miR-137 was a hypoxia-responsive axis involved in the astrocyte-microglia crosstalk under acute I/R injury. Over-expression of lncRNA GAS 5 inhibited miR-137 expression; downregulated miR-137 promoted the INPP4 expression, thereby inhibiting PI3K/Akt pathway activation and leading to apoptosis and inflammation.

**Figure 9:**
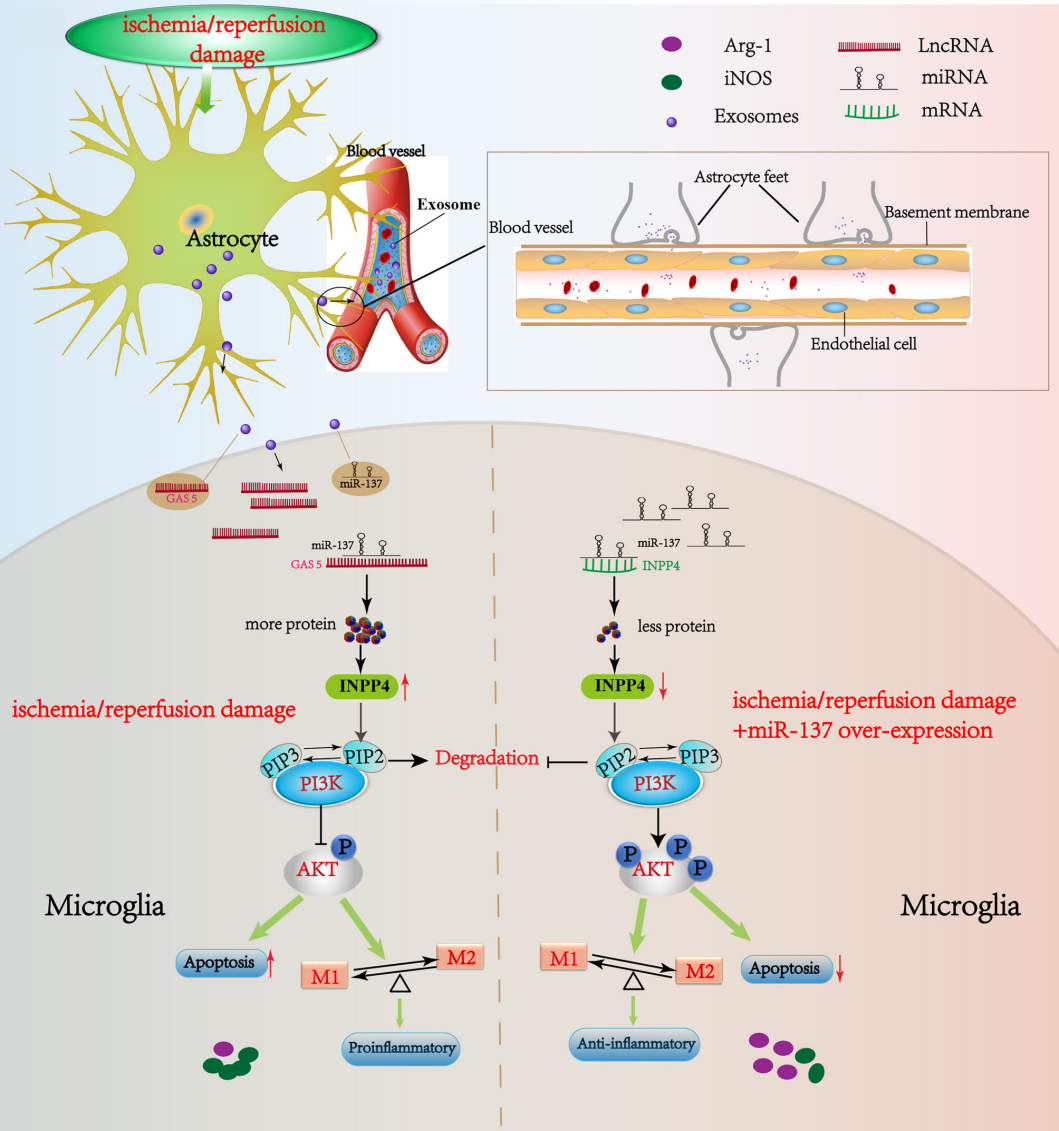
Schematic diagram of this study. LncRNA GAS5/miR-137 is a hypoxia-responsive axis involved in the astrocyte–microglia crosstalk in response to acute systemic ischemia/reperfusion (I/R) injury. The mRNAs (INPP4B) competitively bind miR-137 with lncRNAs GAS5, which forms a ceRNA network to regulate the apoptosis and inflammation phenotype of microglia through PI3K/AKT signal phosphorylation. The acute I/R injury stimulates the release of astrocytes-derived exosomes, and then exosomes are uptaken by microglia. LncRNA GAS5 and miR-137 mutually regulated each other negatively. If miR-137 is over-expressed, microglial apoptosis can be partially rescued and transformation to pro-inflammation phenotype is suppressed *via* regulating INPP4B in CA/CPR and OGD/R. Moreover, we speculate that the significant changes in the lncRNA GAS5/miR-137 in the blood of CA/CPR patients are released by astrocytes through blood–brain barrier (BBB), or by the other organs such as heart, kidney and liver, into the blood under the systemic I/R stimulation.

In animal experiments, we repeatedly observed the damage of the blood-brain barrier (BBB) in CA mice. However, the BBB damage in CA mice was not as obvious as in stroke mice (28, 29). Therefore, we are, for now, unable to determine whether the significant changes in the lncRNA GAS5/miR-137 in the blood of CA/CPR patients are released by astrocyte through BBB, or by the other organs such as heart, kidney and liver, into the blood under the systemic I/R stimulation. Even so, our result showed that lncRNA GAS5/miR-137, a newly identified hypoxic responsive axis, plays a key role in CA/CPR, an acute systemic I/R injury (**Figure 9**). The axis has great potential to serve as a molecular target for the treatment of CA/CPR associated multiple organ dysfunction.

Briefly in review, our commitment to microarray sequencing analyses will dissect the molecular events that result from vesicles trafficking in acute I/R microenvironment and lead to the identification of more suitable therapeutic targets. While the specifics are intriguing, and the research workload is huge, our substantial research explores a new form of intercellular communication and opens up prospects for the broader exosomal research for CA/CPR associated neuro-injury. However, our research has several limitations (1): We used an OGD/R model rather than CA/CPR mice model in the study of the lncRNA GAS5/miR-137 involved in the astrocyte–microglia crosstalk. We did not include lncRNA GAS 5 on and off function research in the CA/CPR mice model, given that the grouping would be too complicated (2). At the stage of preliminary experiment, we designed miR-137 on and off function research in the CA/CPR mice model, but few mice survived the day 1 after CA/CPR surgery in the miR-137 antagomir infusion group. Intriguingly, there was an obvious improvement in overall survival rate in the CA/CPR group than in the miR-137 agomir group (46.2% *vs.* 75%, *p* = 0.1269, **Supplementary Figure 1**), but no significant difference existed between two groups (3). Due to ethical considerations, we did not obtain specimens of brain tissues, cerebrospinal fluid, or solid organs from CA/CPR patients, regardless of the outcome of CA/CPR patients.

## Conclusion

Based on the bioinformatic analysis, microarray results, *in vitro and in vivo* data, we were led to theorize that lncRNAGAS5/miR-137 is a hypoxia-responsive axis involved in the astrocyte–microglia crosstalk and it inhibits PI3K/Akt signaling by modulating INPP4B. Knockout of lncRNAGAS5 or over-expression of miR-137 activated the PI3K/Akt phosphorylation and inhibited apoptosis and neuro-inflammation under the acute systemic ischemia/reperfusion injury.

## Data Availability Statement

The original contributions presented in the study are included in the article/**Supplementary Material**. Further inquiries can be directed to the corresponding author.

## Ethics Statement

The studies involving human participants were reviewed and approved by the Clinical Research Ethics Committee of Renmin Hospital of Wuhan University. The patients/participants provided their written informed consent to participate in this study. The animal study was reviewed and approved by the Laboratory Animal Welfare & Ethics Committee of Renmin Hospital of Wuhan University.

## Author Contributions

XX, ZL and LJ designed the study, revised and finalized the manuscript. WJ, XT, and KL performed the experiments *in vitro* and *in vivo*. JJ and YL collected and analyzed the clinical data. LX and JG performed statistical analysis, as well as the revision of the manuscript. All authors contributed to the article and approved the submitted version.

## Funding

This work was supported by a grant from National Key Research and Development Plan (2020YFC0846600), a grant from the National Natural Science Foundation of China (NSFC 81372020), a grant from Renmin Hospital of Wuhan University (RMYD 2018Z15) and a grant of Young Talent Physician Training Project in Wuhan City (2014ZX0001).

## Conflict of Interest

The authors declare that the research was conducted in the absence of any commercial or financial relationships that could be construed as a potential conflict of interest.

## Publisher’s Note

All claims expressed in this article are solely those of the authors and do not necessarily represent those of their affiliated organizations, or those of the publisher, the editors and the reviewers. Any product that may be evaluated in this article, or claim that may be made by its manufacturer, is not guaranteed or endorsed by the publisher.

## Supplementary Material

Supplementary Figure 1 Survival rate in the different groups. The survival curves were analyzed using the Log-rank (Mantel-Cox) test.

Supplementary Figure 2 Representative gating strategy for flow cytometric analysis. The above row demonstrated microglia and subpopulation in the brain of sham surgery. The following row demonstrated microglia and subpopulation (activated microglia/macrophages, resting microglia) in the brain on day 3 after CA/CPR.

Supplementary Chart 1
**(A)** cell experimental design and grouping. **(B)** mice model experimental design and grouping.

Supplementary Table 1 Baseline characteristics of cardiac arrest patients.

Supplementary Table 2 Primer sequences for qPCR.

Supplementary Table 3 Antibodies for flow cytometry analysis, immunofluorescence staining, and Western blot.

## Abbreviations

LncRNAs, long non-coding RNAs; miRNA, microRNA; miR-137, microRNA-137; LncRNA GAS5, LncRNA growth arrest specific transcript 5; INPP4B, inositol polyphosphate 4-phosphatases B; PI3K, phosphoinositide 3-kinase; CA/CPR, cardiac arrest/cardiopulmonary cerebral resuscitation; PCAS, Post Cardiac Arrest Syndrome; OGD/R, oxygen glucose deprivation/reoxygenation; ROSC, return of spontaneous circulation; I/R, ischemia/reperfusion; BBB, blood–brain barrier; TEM, transmission electron microscopy.
