# Supplementary figures and images for: LncRNA GAS5/miR-137 Is a Hypoxia-Responsive Axis Involved in Cardiac Arrest and Cardiopulmonary Cerebral Resuscitation
Source: Front Immunol. 2022 Jan 11;12:790750. doi: 10.3389/fimmu.2021.790750 (PMC8787067; doi:10.3389/fimmu.2021.790750)

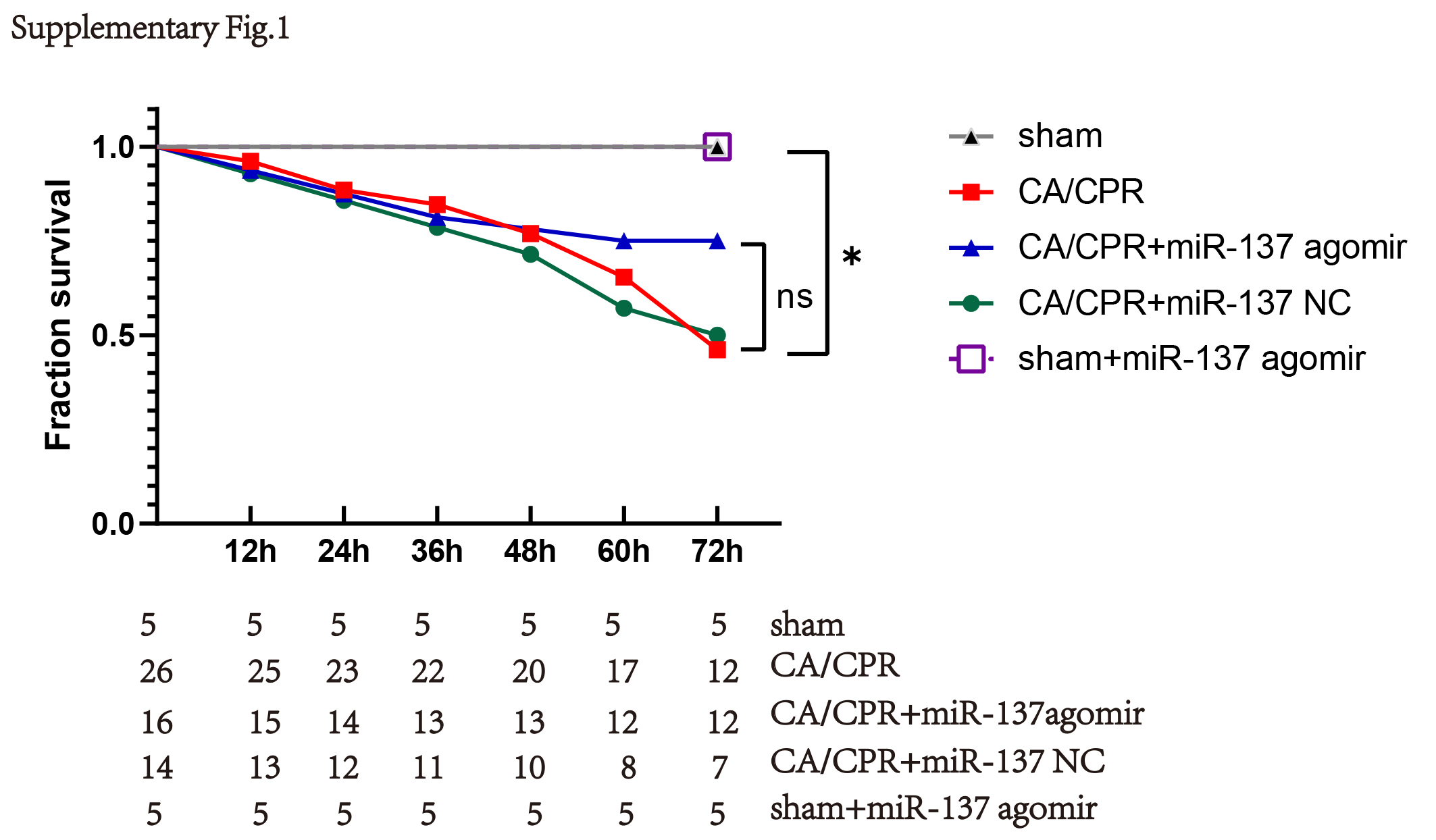

Supplement: Supplementary Figure 1 — Survival rate in the different groups. The survival curves were analyzed using the Log-rank (Mantel-Cox) test. [file Image_1.jpeg]

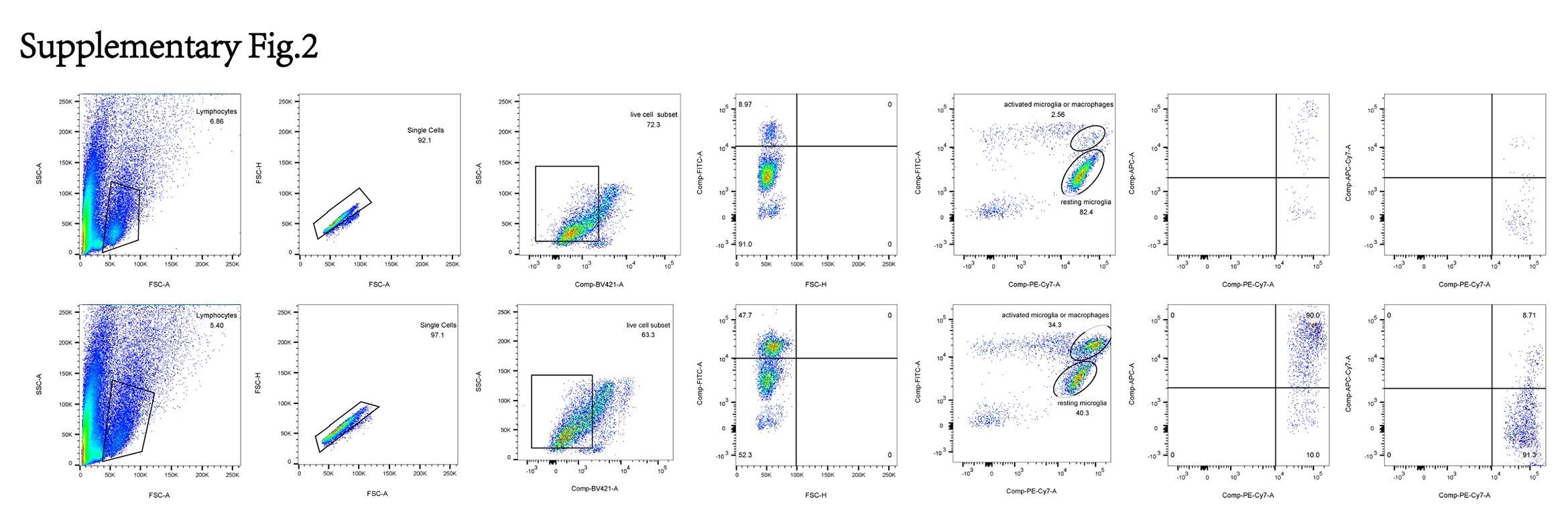

Supplement: Supplementary Figure 2 — Representative gating strategy for flow cytometric analysis. The above row demonstrated microglia and subpopulation in the brain of sham surgery. The following row demonstrated microglia and subpopulation (activated microglia/macrophages, resting microglia) in the brain on day 3 after CA/CPR. [file Image_2.jpeg]

A

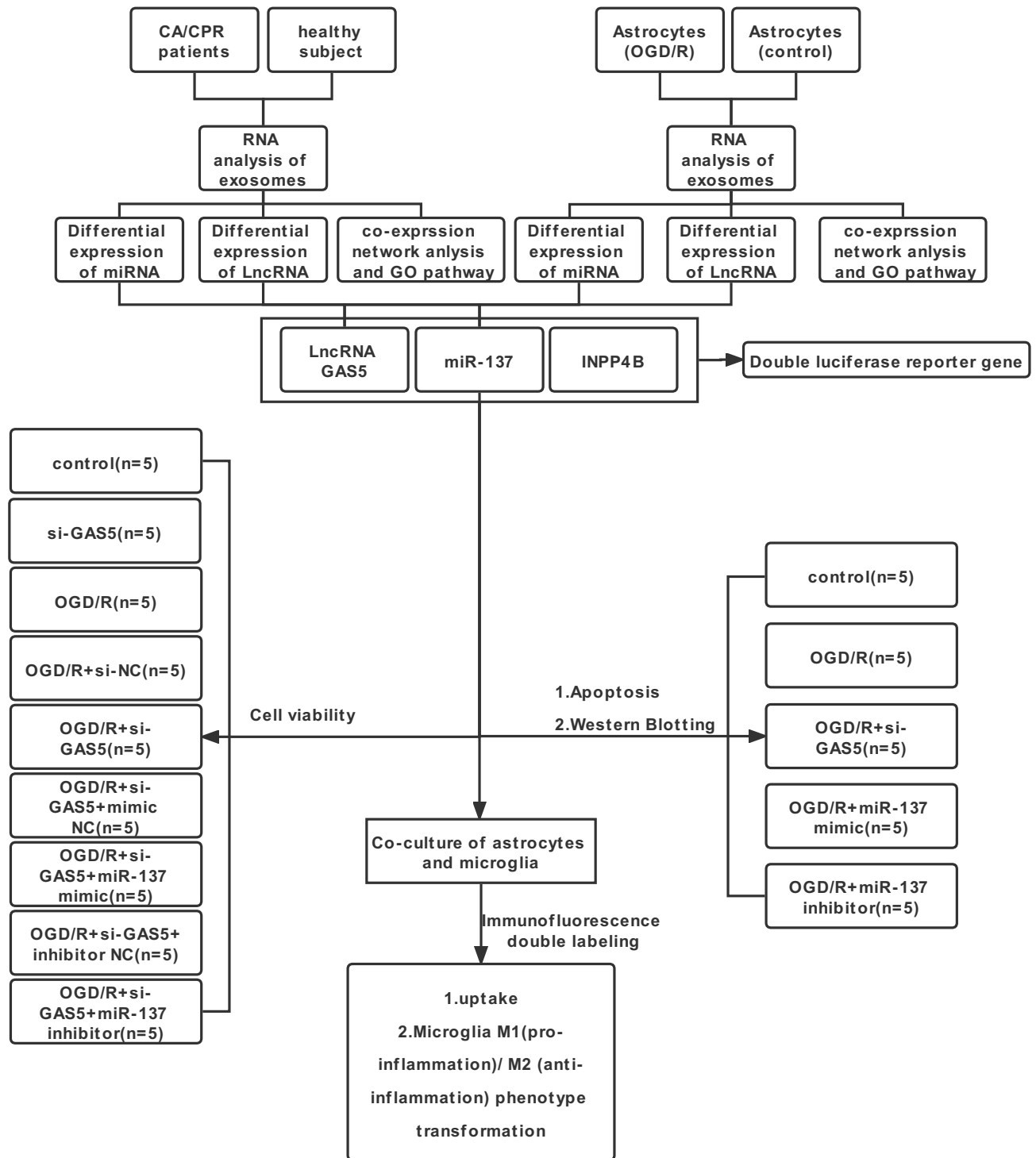

B

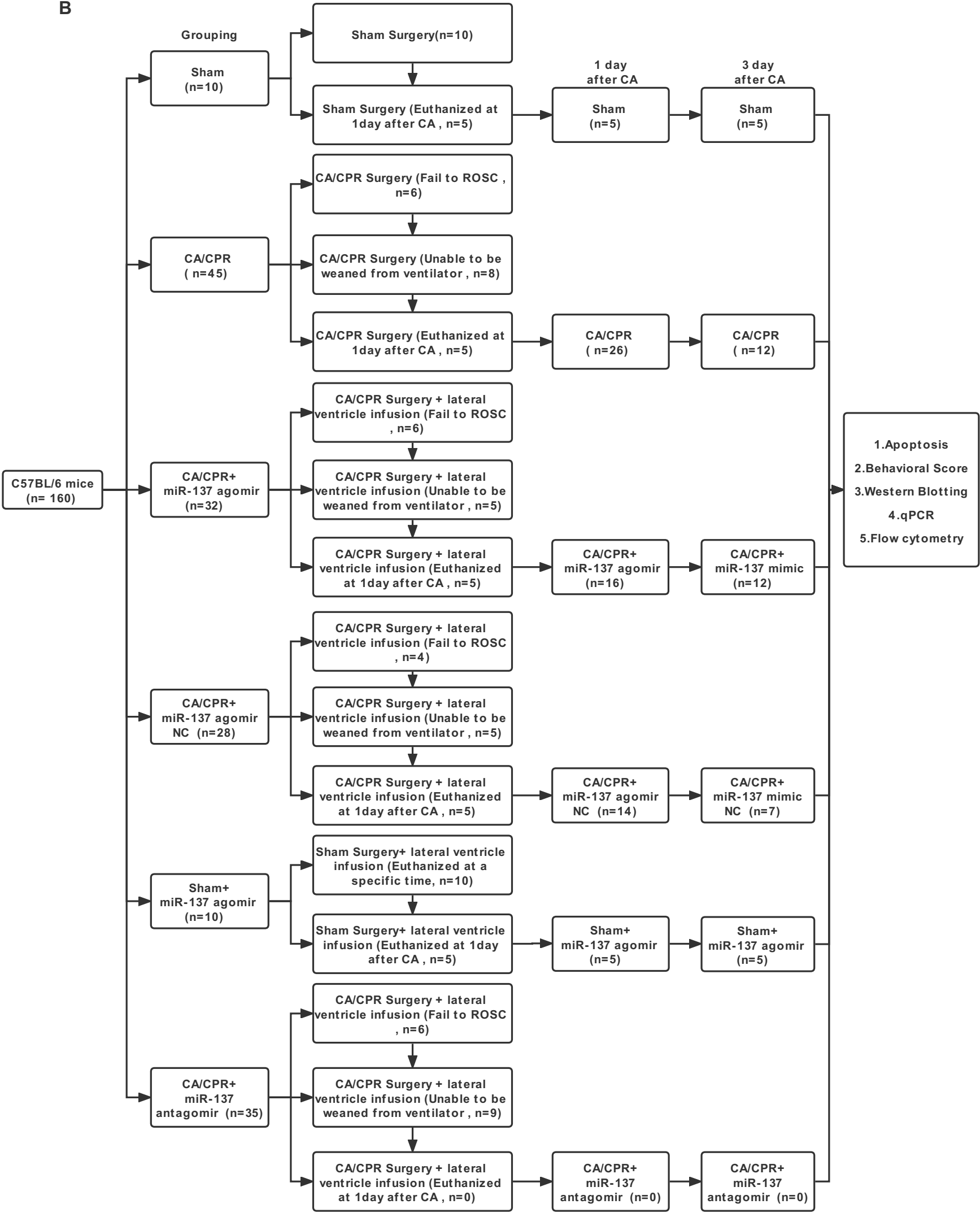

Supplement: Supplementary Chart 1 — (A) cell experimental design and grouping. (B) mice model experimental design and grouping. [file DataSheet_1.pdf]
