# Supplementary material for: LncRNA GAS5/miR-137 Is a Hypoxia-Responsive Axis Involved in Cardiac Arrest and Cardiopulmonary Cerebral Resuscitation
Source: Front Immunol. 2022 Jan 11;12:790750. doi: 10.3389/fimmu.2021.790750 (PMC8787067; doi:10.3389/fimmu.2021.790750)
Supplement: Supplementary Table 1 — Baseline characteristics of cardiac arrest patients. [file Table_1.docx]

**Supplementary Table 1. Baseline characteristics of cardiac arrest patients**

| **Patient**  **ID** | **No.**  **hospitalized** | **Gender** | **Age**  **(year)** | **Cause of Cardiac Arrest** | **Medical history** | **In-hospital CA or Out of hospital CA** | **ECG**  **manifestation** | **Duration from CA**  **to ROSC (min)** | **Adrenaline dosage (mg)** | **Stay in hospital (day)** | **Prognosis** |
| --- | --- | --- | --- | --- | --- | --- | --- | --- | --- | --- | --- |
| 01 | 10136021 | male | 19 | Hyperkalemia | hepatic metastasis of colonic carcinoma Sepsis | Out of hospital CA | Ventricular fibrillation | Unknown | 5 | 41 | **death** |
| 02 | 10121361 | male | 68 | Sepsis | Sepsis | In-hospital CA | Cardiac arrest | 9 | 8 | 73 | **death** |
| 03 | 10136565 | male | 47 | Cerebral hernia | Postoperative Meningioma | In-hospital CA | Unknown | 22 | 0 | 18 | **death** |
| 04 | 10146887 | male | 67 | Hemorrhagic shock | Aortic dissection | In-hospital CA | Unknown | 0 | 1 | 11 | **death** |
| 05 | 10097398 | female | 83 | Acute heart failure | Hypertension  Renal insufficiency | Out of hospital CA | Cardiac arrest | 15 | 4 | 2 | **death** |
| 06 | 10082932 | female | 91 | Acute stroke | Hypertension  Diabetes | In-hospital CA | Cardiac arrest | 15 | 3 | 8 | **death** |
| 07 | 10077375 | male | 64 | Acute heart failure | Heart failure  Diabetes | Out of hospital CA | Ventricular fibrillation | 20 | 10 | 1 | **death** |
| 09 | 10064603 | male | 68 | Cerebral hernia | Hypertension  Chronic alcoholism | Out of hospital CA | Cardiac arrest | 15 | 6 | 2 | **death** |
| 10 | 10106621 | female | 19 | Subarachnoid hemorrhage | - | Out of hospital CA | Cardiac arrest | 15 | 5 | 2 | **death** |
| 11 | 10116219 | male | 48 | Hypokalemia | Hypokalemia  Depression | Out of hospital CA | Cardiac arrest | 19 | 0 | 11 | **death** |
| 12 | 10161040 | male | 20 | Hypokalemia | - | Out of hospital CA | Cardiac arrest | 20 | 2 | 10 | **recovery** |
| 13 | 10101556 | male | 71 | Respiratory failure | Coronary Artery Disease  Diabetes | Out of hospital CA | Cardiac arrest | 25 | 4 | 7 | **death** |
| 14 | 10109533 | female | 80 | Acute heart failure | Hypertension  Diabetes  Heart failure | Out of hospital CA | Cardiac arrest | 20 | 5 | 14 | **death** |

**Supplementary Table 2. Primer sequences for qPCR**

| **Gene** |  | **Primer sequence (5’-3’)** |
| --- | --- | --- |
| TNF-α | Forward | TGCCTATGTCTCAGCCTCTTC |
|  | Reverse | CTCCTCCACTTGGTGGTTTG |
| IL-1β | Forward | CAGGCAGGCAGTATCACTCA |
|  | Reverse | GCCCAAGGCCACAGGTAT |
| IL-4 | Forward | GTAGGGCTTCCAAGGTGCTTC |
|  | Reverse | CATGATGCTCTTTAGGCTTTCCAG |
| IL-10 | Forward | ACCTGGTAGAAGTGATGCCC |
|  | Reverse | ACACCTTGGTCTTGGAGCTT |
| LncRNAGAS5(mouse) | Forward | GGATAACAGAGCGAGCGCAAT |
|  | Reverse | CCAGCCAAATGAACAAGCATG |
| miR-137-5p(mouse) | Forward | TTATTGCTTAAGAATACGCG |
|  | Reverse | TCGTATCCAGTGCAGGGTC |
| INPP4A(mouse) | Forward | TAAGCTGAGGAACTGCCTGCATGA |
|  | Reverse | TGGA AGTGGCCTGAGTGACTTTGA |
| INPP4B(mouse) | Forward | GTGGCGGCAA CAATGATGGAGAAA |
|  | Reverse | TACGCAAGTTCCTGAAGGAGCACA |
| GAPDH(mouse) | Forward | TGGAGAAACCTGCCAAGTATG |
|  | Reverse | ATGTAGGCCATGAGGTCCAC |
| U6(mouse) | Forward | GCTCGCTTCGGCAGCACAT |
|  | Reverse | AAAATATGGAACGCTTCACG |
| LncRNAGAS5(human) | Forward | CTTCTGGGCTCAAGTGATCCT |
|  | Reverse | TTGTGCCATGAGACTCCATCAG |
| miR-137-5p(human) | Forward | GTGACGGGTATTCTTGGGT |
|  | Reverse | GACTACGCGTATTCTTAAGCAA |
| INPP4B(human) | Forward | AGAGCTTTAGATTGCATGAGAAGAGA |
|  | Reverse | GGGAGCCCTCTTTGCTTTTA |
| U6(human) | Forward | CTCGCTTCGGCAGCACA |
|  | Reverse | AACGCTTCACGAATTTGCGT |
| GAPDH(human) | Forward | TGACAACTTTGGTATCGTGGAAGG |
|  | Reverse | AGGCAGGGATGTTCTGGAGAG |

**Supplementary Table 3. Antibodies for flow cytometry analysis, immunofluore- scence staining, and Western blot**

| **Applications** | **Antibody (Clone)** | **Cat. No.** | **Sources** |
| --- | --- | --- | --- |
| Flow cytometry | FITC anti-CD45(30-F11) | 103107 | BioLegend |
|  | PE anti-CD45(30-F11) | 103105 | BioLegend |
|  | PE/Cy7 anti-CD11b (M1/70) | 101215 | BioLegend |
|  | APC anti-F4/80(QA17A29) | 157305 | BioLegend |
|  | APC/Cy7 anti-Ly6G (1A8) | 127624 | BioLegend |
|  | FITC anti-Ly6G (1A8) | 127605 | BioLegend |
|  | Anti-Mouse CD16/CD32 (Mouse BD Fc Block™) Clone:2.4G2 | 553141 | BD  Bioscience |
|  | LIVE/DEAD™ Fixable Violet Dead Cell Stain | L34964 | Invitrogen |
|  | APC Annexin V Apoptosis Detection Kit with 7-AAD | 640930 | BioLegend |
|  | Alexa Fluor® 488 anti-GFAP (SMI 25) | 837507 | BioLegend |
|  | IBA1 polyclonal antibody(unconjugated) | PA5-27436 | Invitrogen |
|  | Alexa Fluor 488 secondary antibody, Rabbit anti-Mouse IgG (H+L) | A27023 | Invitrogen |
| Immunofluorescence staining | Anti-Rabbit CD9 | DF6565 | Affinity |
|  | Anti-Rabbit iNOS | AF0199 | Affinity |
|  | Anti-Rabbit Arg1 | DF6657 | Affinity |
|  | Anti-GFAP Mouse mAb | GB12096 | Servicebio |
|  | Anti-Iba1 Mouse mAb | GB12105 | Servicebio |
|  | Anti-CD63 Rabbit pAb | GB11620 | Servicebio |
| Western blot | β-actin | A3854 | Sigma |
|  | INPP4B | PA5-72878 | Invitrogen |
|  | Rabbit monoclonal antibody[EPR18702] to PI3 Kinase p85 alpha | ab191606 | abcam |
|  | Rabbit polyclonal to pan-AKT | ab8805 | abcam |
|  | Rabbit polyclonal antibody to PI3 Kinase p85 alpha (phospho Y607) | ab182651 | abcam |
|  | Rabbit polyclonal to AKT (phospho T308) | ab38449 | abcam |
